# Circulating monocytes in acute pancreatitis

**DOI:** 10.3389/fimmu.2022.1062849

**Published:** 2022-12-12

**Authors:** Shiyu Liu, Peter Szatmary, Jing-wen Lin, Qiqi Wang, Robert Sutton, Lu Chen, Tingting Liu, Wei Huang, Qing Xia

**Affiliations:** ^1^ West China Centre of Excellence for Pancreatitis, Institute of Integrated Traditional Chinese and Western Medicine, West China-Liverpool Biomedical Research Centre, West China Hospital, Sichuan University, Chengdu, China; ^2^ Liverpool Pancreatitis Research Group, Liverpool University Hospitals NHS Foundation Trust and Institute of Systems, Molecular and Integrative Biology, University of Liverpool, Liverpool, United Kingdom; ^3^ State Key Laboratory of Biotherapy, West China Hospital, Sichuan University and Collaborative Innovation Center for Biotherapy, Chengdu, China; ^4^ Institutes for Systems Genetics & Immunology and Inflammation, Frontiers Science Center for Disease-related Molecular Network, West China Hospital, Sichuan University, Chengdu, China

**Keywords:** acute pancreatitis, monocytes, immunity, inflammation, animal models, lipotoxicity, obesity, hypertriglyceridemia

## Abstract

Acute pancreatitis is a common gastrointestinal disease characterized by inflammation of the exocrine pancreas and manifesting itself through acute onset of abdominal pain. It is frequently associated with organ failure, pancreatic necrosis, and death. Mounting evidence describes monocytes - phagocytic, antigen presenting, and regulatory cells of the innate immune system - as key contributors and regulators of the inflammatory response and subsequent organ failure in acute pancreatitis. This review highlights the recent advances of dynamic change of numbers, phenotypes, and functions of circulating monocytes as well as their underling regulatory mechanisms with a special focus on the role of lipid modulation during acute pancreatitis.

## 1 Introduction

Acute pancreatitis (AP) is a common gastrointestinal disease with a globally rising incidence (1). Patients present with very acute onset of severe abdominal pain often necessitating urgent hospital admission (2), an event which is thought to coincide with the time of initial pancreatic injury. After the initial insult by pancreatitis toxins, injured pancreatic acinar cells release cytokines, chemokines, cellular components, and neuropeptides to promote inflammation (3–5). Immune cells are closely related with the systemic response to pancreatic injury, thereby contributing substantially to disease severity (6–9). Neutrophils and monocytes are recruited to the pancreas during early stages of AP, followed by dendritic cells (DCs), mast cells, and T cells (8, 10). Neutrophils promote inflammation and tissue damage in AP through further secretion of cytokines and chemokines, as well as through generation of reactive oxygen species (ROS) (11), enhancement of nicotinamide adenine dinucleotide phosphate oxidase (12) and granule enzymes activity (13, 14), release of neutrophil extracellular traps (15), and promotion of trypsinogen activation (16). The critical role of neutrophils in the pathogenesis of AP has been recently reviewed (17, 18). There is some limited evidence to suggest that DCs possess both protective (19) and pro-fibroinflammatory roles (20), while mast cells are often reported to contribute to deleterious outcomes of AP (21, 22). The role of T cells in AP is complex, with AP severity correlating with T cell infiltration and being regulated by the balance of Th1/Th2 and Treg/Th17 cells (23, 24).

Monocytes/macrophages are also widely accepted as the key contributors and main inflammatory cell population during initiation and progression of systemic inflammation in AP (7, 25). Monocyte-derived macrophages form the bulk of the macrophage population in inflamed pancreatic tissue (26) and while tissue macrophages in AP have been extensively reviewed (25, 27), the role of circulating monocytes remains largely neglected. This review aims to detail the current understanding of circulating monocytes in AP.

## 2 Monocytes

Monocytes are short-lived mononuclear phagocytes characterized by kidney-shaped nuclei, and are the largest leucocyte subtype in blood, making up about 5-10% of circulating blood leucocytes (28, 29). They are a crucial component of the innate immune system and serve as phagocytes and antigen-presenting cells as well as having a critical role in orchestrating the immune response to infection and inflammation (30–34). Traditionally, monocytes were regarded as transitional cells – non-terminally differentiated, circulating forms of macrophages or DCs. Increasingly, distinct functions of heterogeneous subsets of monocytes with their own transcriptional profiles and phenotypes are being described in inflammatory and autoimmune diseases (30, 35).

Monocytes develop primarily in the bone marrow (BM) in a highly regulated manner (33). Spleen is a primary site of extramedullary monocytopoiesis and contains a monocyte reservoir with over one million monocytes (36, 37). The common monocyte progenitor population is identified to be present in BM and spleen, which generates monocytes and monocyte-derived macrophages (38).

There are two main populations of monocytes in human and other species, termed as classical and non-classical monocytes. The proportion of these two subsets are analogous in blood and spleen (37, 39). Classical monocyte fate is regulated by the sequential action of transcription factors including PU.1 (Sfpi1), Irf8, and Klf4 (33, 40). Non-classical monocytes develop from classical monocytes under regulation of Nr4a1 (TR3/Nur77), but the possibility of directly deriving from progenitors in BM cannot be ruled out (33, 40). Intermediate monocytes are thought to be transitional populations between classical and non-classical monocytes (30). The specific markers and distinct functions of the different populations are briefly summarized in **Table 1**.

**Table 1:** Specific immune markers and functions of monocyte subsets.

		Classical monocytes	Non-classical monocytes	Intermediate monocytes	References
Size		18 μM	14 μM	Intermediate	(28, 41)
Approximate proportion of total monocytes		80-95%	2-8%	2-11%	(28)
Approximate lifespan in circulation		1 day	2 days (in mice) or 7 days (in human)	3-5 days	(31, 33, 42)
Functions		Migrating across the endothelium to tissues, maturing into macrophages or remaining as monocytes within tissues	Patrolling vessel walls, recognising and clearing pathogens, supporting endothelial cells, and monitoring vessel integrity	Transitional population between classical and non-classical monocytes	(28, 30)
Identification phenotype	Human	CD14^++^CD16^-^	CD14^+^CD16^++^	CD14^++^CD16^+^	(43)
	Mouse	Ly6C^hi^CD43^low^	Ly6C^low^CD43^hi^	Ly6C^hi/mid^CD43^hi^Treml4^+^	(35, 43)
	Rat	CD43^low^	CD43^hi^CCR2^low^CD62L^low^		(44)
	Pig	CD163^-^	CD163^+^CCR2^low^		(44)
Recruitment		Mediated by CCR2/CCL2	Mediated by CX3CR1/CX3CL1	Mediated by CCR2/CCL2, CX3CR1/CX3CL1	(30, 45)

## 3 Monocytes in AP

During AP, pancreatic acinar and ductal cells are injured in the initial aseptic environment through a series of pathological cellular and molecular events including premature trypsinogen activation, calcium overload, loss of mitochondrial membrane potential, endoplasmic reticulum stress, and impaired autophagy after exposure to pernicious factors such as alcohol metabolites, free fatty acids, bile acids, high intraductal pressure or intraductal acidification (3, 46). Injured cells liberate pro-inflammatory cytokines, chemokines (47), and other inflammatory meditators such as damage-associated molecular patterns (48, 49) locally and into circulation, promoting leucocytes infiltration and activation which exacerbates pancreatic injury, systemic inflammation, and organ failure (4).

**Figure 1:**
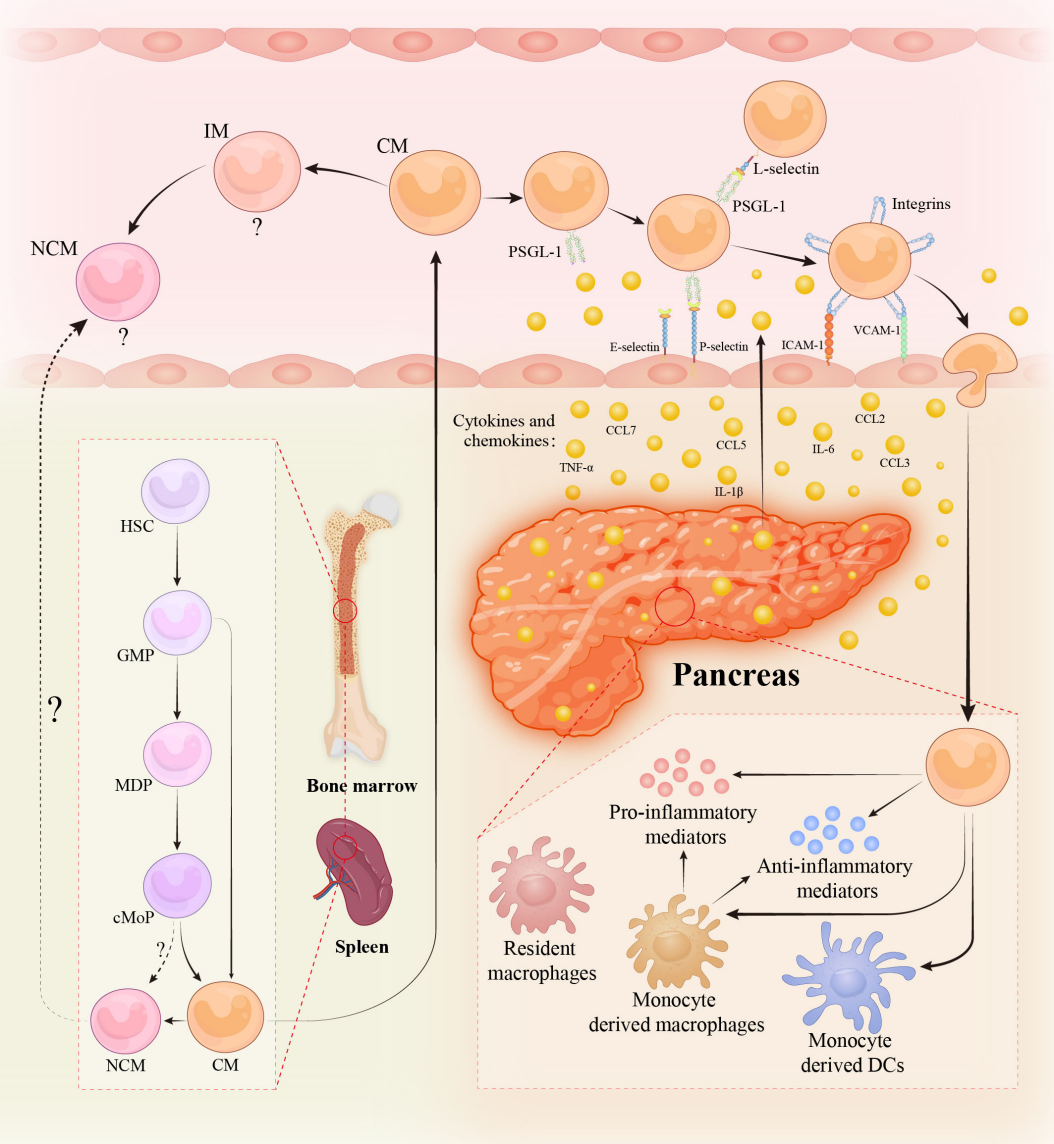
Monocytes in acute pancreatitis. Monocytes are continuously produced in bone marrow (BM) and spleen from hematopoietic stem cells (HSCs) *via* granulocyte and macrophage progenitors (GMPs), monocyte/dendritic cell (DC) progenitors (MDPs), and common monocyte progenitors (cMoPs) intermediates. During acute and severe inflammation, monocytes might be directly generated from GMPs in response to the need for rapid generation. Non-classical monocytes (NCMs) are derived from classical monocytes (CMs) and might be directly developed from cMoPs. The existence of NCMs in BM/spleen and the regulatory mechanisms of their egress into circulation are disputable and obscure. Intermediate monocytes (IMs) are the transitional cells of CMs and NCMs with distinct functions. The role of NCMs and IMs in acute pancreatitis (AP) is still vague. During AP, pancreatic acinar and ductal cells are injured and liberate damage-associated molecular patterns, cytokines, chemokines, and various pro-inflammatory mediators, leading to the recruitment of circulating monocytes to the inflamed pancreas. Monocytes migration across the endothelium requires a series of sequential adhesive interactions between monocytes and endothelial cells mediated by the indicated endothelial adhesion molecules and monocyte selectin and integrin ligands. When monocytes influx into the pancreas, they either develop into monocyte-derived macrophages/DCs or maintain monocyte-state. The infiltrated monocytes and monocyte-derived macrophages exhibit heterogeneous phenotypes such as pro-inflammatory or anti-inflammatory activities, depending on the environmental cues. Abbreviations: PSGL-1, P-selectin glycoprotein ligand 1; ICAM-1, intercellular adhesion molecule-1; VCAM-1, vascular cellular adhesion molecule-1.

Among inflammatory mediators, the released CC-chemokines including CC-motif chemokine ligand 2 (CCL2; or monocyte chemoattractant protein-1, MCP-1), CCL3 (macrophage inflammatory protein-1α, MIP-1α), CCL5 (RANTES), and CCL7 (MCP-3) give rise to recruitment of circulating monocytes to the inflamed pancreas (25, 50). CCL2 is a canonical chemokine produced by stromal cells or immune cells in acute inflammation, dimerizes, and binds to extracellular matrix glycosaminoglycans forming a stable gradient to attract CCR2^+^ inflammatory monocytes (7, 50). CCL2 levels were increased in pancreatic, systemic, lung, intestinal, and urinary samples and correlated with severity in experimental and clinical AP (51–57). The infiltrating monocytes become activated and gain distinct immune phenotype in response to the environmental cues, which afterwards either serve as monocyte reservoirs with proliferative activity or further differentiate into macrophages or DCs (33) (**Figure 1**). The key findings of major studies on monocytes in experimental and clinical AP are summarized in **Supplementary Table 1** and **Supplementary Table 2** respectively.

### 3.1 Dynamic changes of monocyte numbers

Human pancreas is often difficult to obtain for mechanistic studies. Therefore, experimental animal models have been widely employed to investigate the pathobiology of AP for over a century and a half (58). Of the experimental animals, mice and rats are preferred as the mammalian pancreas differs markedly from non-mammals in structure and function and due to the relative ease of genetic manipulation in these species (59). Repetitive administration of caerulein, a cholecystokinin analogue, causes clinical, biochemical and histopathological changes in rodent pancreas similar to those seen in human AP (60). Caerulein-induced AP (CER-AP) is the most popular experimental model, as it is easily reproducible, simple to perform and the severity can be adjusted by different dosing regimens (58). Further, a septic condition with profound systemic inflammation and organ failure can be produced by superimposing a single injection of lipopolysaccharide (LPS) on CER-AP (CER/LPS-AP) (58). Choline-deficient ethionine-supplemented diet and intraperitoneal administration of L-arginine are other less-invasive ways to induce AP (CDE-AP and ARG-AP, respectively) (58, 61). These AP models are often associated with significant systemic inflammation, organ failure and a certain rate of mortality, thereby mirroring the pathobiology of SAP (58). Both ductal infusion of bile acid, sodium taurocholate induced-AP (NaTC-AP) and pancreatic duct ligation-induced AP (PDL-AP) are designed to mimic human biliary pancreatitis and have features of clinical SAP (62, 63).

Observational studies looking at monocyte subtypes in circulation or within the pancreas in association with different stages of AP as well as its severity, together with depletion studies in experimental models form the backbone of our understanding of the role of monocytes in AP. Most detailed studies of the timeline of monocyte response during AP come from experimental models, where the time of disease onset is carefully controlled.

In mice with CER-AP, the number of circulating monocytes peaked at 12 h and pancreatic monocytes at 24 h, both returning to baseline during the recovery phase (by 168 h) (64). Following NaTC-AP and in PDL-AP in rats, monocyte numbers in peripheral blood were significantly elevated from 30 min and 6 h respectively (65, 66). An increased frequency of monocytes was observed both in circulation and in the pancreas at 72 h in mice with CDE-AP but this was not statistically significant in circulation (64). In a similar manner, peripheral monocytes were elevated in AP patients on admission, especially those who had more severe clinical phenotype (67–69). Monocyte levels remained elevated up to one week after admission (67–69). Clinically, lymphocyte-to-monocyte ratio (LMR) could serve as one of the predictive indices for persistent organ failure and mortality (70–75). Compared with mild AP (MAP; no organ failure, no local or systemic complications) or moderately severe AP (MSAP; transient organ failure or local or systemic complications) patients, those with severe AP (SAP; persistent organ failure) as defined by the revised Atlanta classification (76) had a lower LMR (71, 73, 74, 77, 78). Paradoxically, SAP patients with uncertain time of disease onset had reduced lymphocyte and monocyte counts at the early stage of their ICU stay, perhaps indicating an immune anergy later in severe disease, which recovered to normal on day 7 (79). Depletion of monocytes/macrophages in circulation and tissue by clodronate liposomes reduced disease severity in CER-AP mice and NaTC-AP rats (80, 81) if used prior to AP induction. The method was also used to describe the dynamic phenotypes (pro-inflammatory and restorative) during CER-AP and recovery (26, 82). Conditional depletion of monocytes, achieved by administration of diphtheria toxin (DT) to mice with transgenic expression of the human diphtheria toxin receptor [DTR] coupled to the CD11b promoter (CD11b-DTR), reduced pancreatic edema and necrosis in CER-AP and NaTC-AP (83). Targeting the CCL2 axis with genetic ablation or pharmacological inhibition reduced the number of monocytes/macrophages in the pancreas and ameliorated the severity of CER-AP, ARG-AP, and NaTC-AP models (84–86).

It was first demonstrated in the 1970s that monocytes increased proliferative activity in BM during inflammation, resulting in monocytosis (87). Intriguingly, cholesterol metabolism plays a crucial role in regulating proliferation of hematopoietic stem and progenitor cells (88). And hypercholesterolemia is reported to increase circulating monocytes *via* promoting hematopoietic stem and progenitor cell proliferation in BM and seed in spleen (88). However rapid changes in the number of circulating leucocytes are a function of release of cells from storage pools, rather than *de novo* synthesis of cells. Classical monocytes have been shown to egress from BM in a CCR2-dependent manner. CCL2 and CCL7 are ligands for CCR2 and help maintain the stable levels of circulating monocytes (89). Whether non-classical monocytes exist in the BM and the mechanism of them exiting the BM are as yet unclear. The spleen contains a mobilizable monocyte reservoir in the subcapsular red pulp, with morphology and transcriptomes of Ly6C^high^ monocytes indistinguishable from those in blood (39, 90). Angiotensin II or CCR2 signaling is reported to induce monocytes egress from the spleen to the sites of injury and tumor, such as myocardial infarction and lung adenocarcinoma, resulting in increased circulating monocytes mirrored with reduced splenic monocytes (39, 91). Splenectomy related experiments demonstrate that spleen contributed almost 50% of the monocytes to the infarcted heart and 30% of the monocytes, all the Ly6C^hi^ monocytes to the growing atheroma (92, 93).

In mild CER-AP, increased numbers of peripheral and pancreatic Ly6C^hi^ monocytes were observed concomitant with decreased Ly6C^hi^ monocytes in BM (94). However, the percentage of splenic Ly6C^hi^ monocytes was no different to that of control mice, indicating monocytes were principally mobilized from the BM instead of spleen in this setting (94). In sodium deoxycholate-induced AP rats, splenocytes were recruited to the systemic circulation and resulted in splenic atrophy (95). Splenectomy after AP induction in this model reduced serum amylase, inflammatory cytokine levels and pancreatic histological injury (96). The precise role of splenic monocytes in AP remains unclear.

### 3.2 Phenotypic changes of monocyte immune markers

Heterogeneity of monocyte subsets is increasingly becoming apparent (33). However, studies addressing the role of monocytes in experimental and clinical AP principally pertain to classical monocytes. The involvement of non-classical, or indeed intermediate monocytes in the pathogenesis of AP is still unclear.

#### 3.2.1 Classical monocytes

Classical monocytes are described by the expression of the LPS-binding receptor CD14 and the absence of the FcγRIII (CD16) receptor. In AP patients, the proportion and absolute numbers of circulating classical monocytes (CD14^++^CD16^-^) increase compared to healthy controls and correlate with escalating severity (MAP < MSAP < SAP) (64, 97–99). Numbers of classical monocytes remain high in SAP patients, whereas in MSAP patients, numbers decrease within one week after AP onset (98, 99). This finding correlates with experimental models of AP, where elevated numbers of equivalent monocytes [CD11b^high^CD11c^-^Gr-1^low^ monocytes/macrophages (86)] in circulation and in the pancreas were shown to correlate with disease severity in CER-AP and ARG-AP in mice (6, 86, 94). In CD11b-DTR mice, administration of DT which conditionally and specifically depletes monocytes/macrophages, abolished the increase of pancreatic Ly6C^hi^ monocytes and was associated with reduced edema and necrosis of the pancreas in CER-AP (83). This process was reversed by adoptive transfer of Ly6C^hi^ monocytes from mice of non-DT-treated CD11b-DTR or from tumor necrosis factor-alpha (*Tnfα*)*^+/+^* donors, but not *Tnfα^-/-^* donors, demonstrating that the pathological role of Ly6C^hi^ monocytes in this setting depends on the TNF-α signaling pathway (83). Ly6C^+^ inflammatory monocytes have also been shown to exhibit significant phenotypic heterogeneity using novel technologies. Seven distinct groups of monocytes could be identified by cell surface markers and cytokine profiles using CyTOF analysis in CER-AP and six in CDE-AP and AP from patient blood samples (64). While these novel subsets of monocytes require validation and further characterization, they offer a significant potential for targeted interventions based on precision cytometry.

#### 3.2.2 Non-classical monocytes

Non-classical monocytes are described as expressing both CD14 and CD16. Studies investigating the role of non-classical monocytes in AP are absent from the published records. In sterile injury including wounds, liver injury and myocardial infarction, a biphasic response was observed with an initial influx of Ly6C^hi^ monocytes producing high levels of interleukin-1 beta (IL-1β) and TNF-α followed by a delayed response from Ly6C^lo^ monocytes/macrophages secreting transforming growth factor-beta (TGF-β) and vascular endothelial growth factor for tissue repair (100–103). Non-classical monocytes also express human leukocyte antigen (HLA)-DR, which is a valuable biomarker of the immune status, whereby a percentage of HLA-DR^+^ monocytes of less than 80% characterizes immunosuppression and below 30% marks immunoparalysis (104). Several studies have demonstrated a downregulation of HLA-DR on monocytes is associated with an increase in AP severity (79, 99, 105–122), and serves as a predictor for SAP, infectious complications, and mortality in AP patients (105, 108–111, 113, 119, 120, 122–125).

Reprograming of Ly6C^hi^ monocytes at the site of inflammation or injury is thought to be more efficient than recruitment of Ly6C^lo^ monocytes to restore homeostasis (82). Cytokines such as IL-4 and IL-10 in the local environment drive the switch of monocytes phenotype from pro-inflammatory Ly6C^hi^CCR2^+^ monocytes to reparative Ly6C^lo^CX3CR1^+^Nr4a1^hi^ monocytes (102). Non-classical monocytes also appeared to be atheroprotective in mice and were associated with vascular repair and improved organ function in kidney and heart models of ischemia reperfusion-induced injury (35). Studies investigating the role of non-classical monocytes in AP are clearly needed.

#### 3.2.3 Intermediate monocytes

Though intermediate monocytes are normally considered to be a transitional state between classical monocytes and non-classical monocytes, they are able to generate reactive oxygen species (ROS) and pro-inflammatory cytokines (TNF-α and IL-1β) in response to LPS, have high proangiogenic capacity and are effective at antigen processing and presentation with strong expression of HLA-DR and Toll-like receptors (TLRs) (126, 127). While a previous study reported that intermediate monocyte (CD14^+^CD16^+^) counts were comparable amongst AP patients and healthy controls within 24 or 72 h of disease onset (97), a more recent study demonstrated that the percentage of intermediate monocytes was reduced in MAP patients compared with healthy controls within 24 h of admission (mean, 2.4% vs. 3.1%, respectively) (127). The reduction of intermediate monocyte numbers was reported to be possibly mediated by a disintegrin and metalloproteinase 17 (ADAM17) and contribute to increased susceptibility to infection, which needs further validation (127). A recent study demonstrated that genetic (*Adam17^ex/ex^* mice, which displayed marked reduction in global ADAM17 expression) or pharmacological blockade of ADAM17 ameliorated the severity of CER-AP mice (17). Considering the ubiquitous expression and multifunctional role of ADAM17 in inflammation (16), it is necessary to figure out the specific pathogenetic effect of ADAM17 on different cells including monocytes so as to exploit a precise therapeutic approach of cell- or tissue-specific ADAM17 inhibition. Overall, the kinetics of circulating intermediate monocytes, their roles in the pathogenesis of AP as well as the underlying mechanisms of alterations in numbers and functions remain to be elusive due to limited studies.

#### 3.2.4 M1 and M2 polarization

M1 and M2 polarization, initially proposed for macrophages and defined by the expression of several cellular markers of polarization, can also be seen in peripheral monocytes in a number of diseases (128–130). M1 monocytes are classically activated and function as pro-inflammatory cells producing inflammatory mediators including TNF-α, IL-1β, IL-6, and IL-12; M2 monocytes are alternatively activated and release anti-inflammatory or regulatory molecules including IL-10 and TGF-β (68, 131). Different subsets of polarization usually dominate at different stages of a disease (68), with the pro-inflammatory M1 polarization state dominating early, followed by a late M2 polarization predominating, dampening inflammation and promoting tissue repair and reorganization.

Numbers of peripheral M1 monocytes (defined by an absence of CD163 and presence of IL-12 or MAC387) and M2 monocytes (defined by CD206 and CD163 positivity) were both significantly elevated in SAP compared to MAP patients or healthy controls (131, 132); and the numbers correlated with Acute Physiology and Chronic Health Evaluation II score (131, 132), one of commonly used composite clinical indices to predict severity of AP (133). By day 3, a higher positive M1:M2 ratio can be observed in the blood of SAP compared with MAP or healthy controls (68). Unfortunately, peripheral monocyte polarization does not appear to be useful in early differentiation of disease severity (i.e., before 48 hours after disease onset) and it remains unclear whether the relationship of disease severity and monocyte polarization is in any way causal.

### 3.3 Function alterations in monocytes

**Figure 2:**
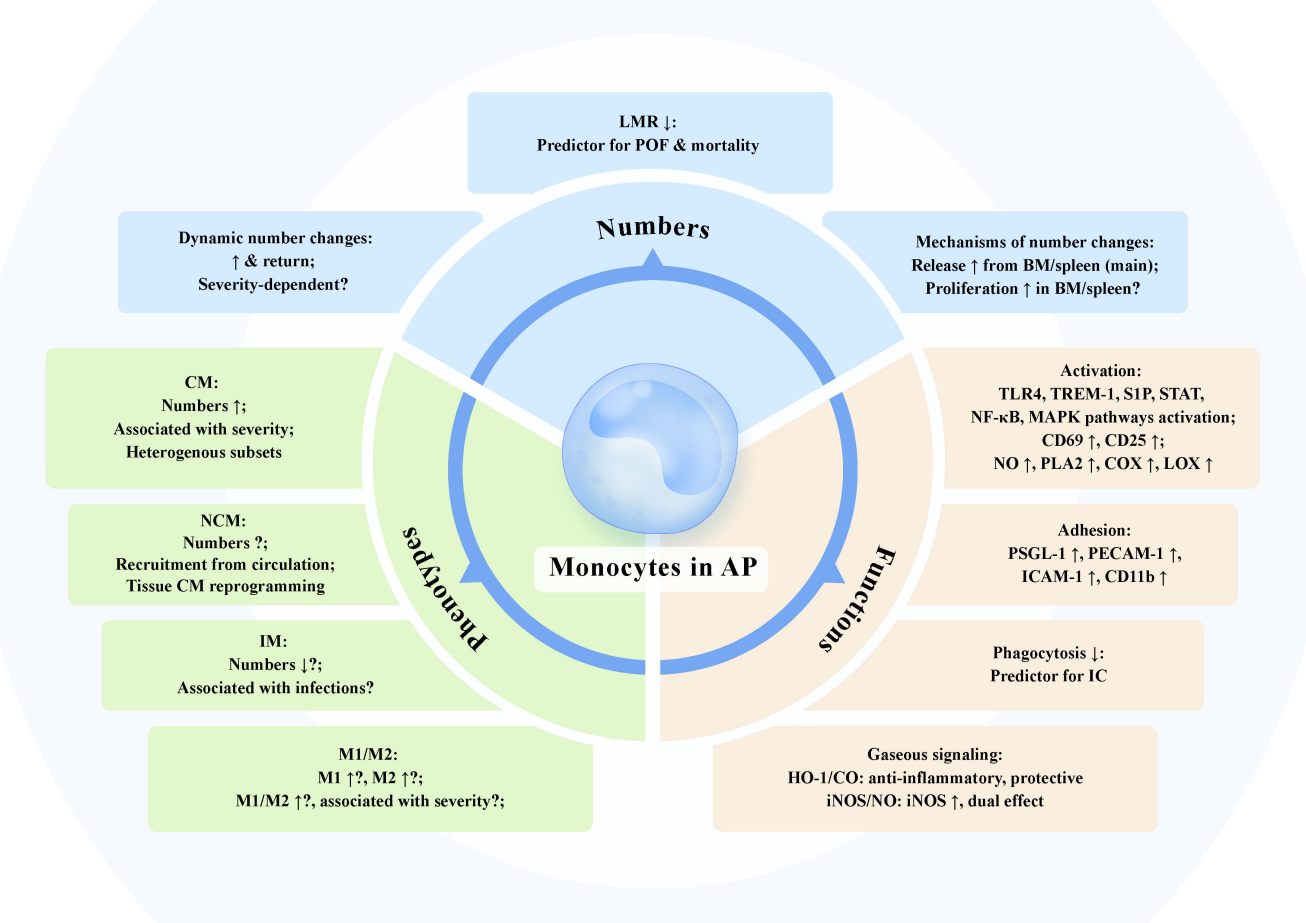
The dynamic changes of monocyte numbers, phenotypes, and functions in acute pancreatitis. *Numbers:* The number of monocytes in circulation and pancreas is dynamically elevated and might return at various timepoints of experimental and clinical acute pancreatitis (AP). The association of the increased number of monocytes with severity of AP has not been fully verified, despite that the increase in monocytes is more pronounced in more severe phenotype in some cases. Lymphocyte-to-monocyte ratio (LMR) is negatively correlated with AP severity and serves as a predictive index for persistent organ failure and mortality. The augmented number of monocytes within a short time is mainly the function of the release from storage pools. The increased proliferation of monocytes and associated progenitors in bone marrow (BM) and spleen may also contribute to the increased circulating monocytes in AP. *Phenotypes:* Circulating classical monocytes (CM) levels are associated with escalating AP severity. Novel heterogeneous subsets of monocytes in AP have been identified. The role of non-classical monocytes (NCM) and intermediate monocytes (IM) in AP is still not known. Decreased number of IM in AP might associate with increased susceptibility to infections. Increased M1 and M2 monocytes together with higher M1/M2 ratio can be observed in AP, which possibly correlate with disease severity. *Functions:* Monocyte activation along with disturbed intracellular multiple signaling profiles and elevated activation markers including CD69 and CD25, leads to massive production of nitric oxide (NO) together with induction of phospholipase A2 (PLA2), cyclooxygenase (COX), and lipoxygenase (LOX). Monocytes in AP exhibit increased adhesion molecules including P-selectin glycoprotein ligand 1 (PSGL-1), platelet endothelial cell adhesion molecule-1 (PECAM-1), and intercellular adhesion molecule 1 (ICAM-1), promoting monocyte adhesion to endothelium and migration to the inflamed pancreas. Impaired phagocytosis of monocytes in AP might be a useful predictor for infectious complication. Heme oxygenase-1 (HO-1)/carbon monoxide (CO) and inducible NO synthase (iNOS)/NO are pivotal gaseous signaling pathways in monocytes of AP. HO-1/CO pathway in monocytes exerts anti-inflammatory effect to dampen AP severity. Increased expression of iNOS in monocytes is observed in AP and the generated NO has dual effect on the production of pro-inflammatory mediators depending on concentrations.

Numerous human and rodent studies have demonstrated the functional variation of monocytes in AP, including alterations in activation, adhesion and phagocytosis. Distinct expression of surface markers and intracellular molecules, and differential change of elaborative regulatory pathways including gaseous signaling pathways in monocytes are associated with specific functional dysregulation such as hyperfunction or anergy, which may together serve as useful tools to assess the immune status during AP (**Figure 2**).

#### 3.3.1 Activation

Monocyte activation takes place in response to activation of TLR4, triggering receptor expressed on myeloid cells-1 (TREM-1) or sphingosine-1-phosphate (S1P) signaling, transduced *via* the signal transducer and activator of transcription (STAT), nuclear factor κB (NF-κB) and mitogen-activated protein kinase (MAPK) pathways (134). Disorders of these signaling profiles in monocytes in AP are briefly summarized in **Table 2**. Evidence for the enhanced activation of monocytes in AP can be seen in both experimental and clinical AP. CD69, the early activation inducer molecule, is a C-type lectin binding protein with a transmembrane and intracellular signaling domain expressed on a wide range of immune cells, including monocytes. Activation of CD69 on monocytes results in nitric oxide production, and induction of phospholipase A2, cyclooxygenase, and lipoxygenase (67). CD25, an IL-2 receptor alpha chain, is expressed on activated T cells, B cells, and monocytes (67). Elevated expressions of both CD69 and CD25 were found in AP patients compared with healthy controls (67, 139).

**Table 2:** Aberrant signaling profiles of monocytes in acute pancreatitis.

Signaling pathways	Roles	Alterations	References
STAT	Mediating gene transcription of inflammatory mediators	(1) Reduced cytokine stimulated pSTAT1, pSTAT3, and pSTAT6 in AP patients than HCs (2) Higher constitutive pSTAT3 in AP patients than HCs (3) Constitutive pSTAT3 levels were early predictor for development of persistent organ failure (AUC 0.725 [0.558-0.893]) and secondary infection (0.662 [0.547-0.776])	(115, 121, 135)
NF-κB	Regulating expression of plentiful genes involved in immunity and inflammation	Reduced NF-κB activation in response to TNF, whole bacteria, and bacteria byproducts in AP patients than HCs	(115, 121, 136)
MAPK	Regulating the adhesion, chemotaxis, and effector functions of monocytes	(1) Upregulated expression of p38 MAPK in NaTC-AP rats than controls (2) AP severity was alleviated by selective MAPK signaling inhibitors	(134)
SphK1/S1P	Regulating critical cellular events including neutrophil priming, cytokines production, and leukocyte chemotaxis Regulating MAPK and NF-κB signaling pathways	(1) Upregulated expression of SphK1 in AP than HCs in the early stage (2) SphK1 levels correlated positively with clinical severity scores in AP patients	(79)
TREM-1	Producing pro-inflammatory mediators and costimulatory molecules	Higher expression of TREM-1 in AP patients than HCs	(137)
TLR4	Triggering pro-inflammatory signaling pathways	(1) Elevated expression of TLR4 expression in AP patients than HCs (2) TLR4 expression correlated with organ failure and mortality	(138)

STAT, signal transducers and activators of transcription; AP, acute pancreatitis; HC, healthy control; AUC, area under the receiver-operating-characteristic curve; NF-κB, nuclear factor-kappa B; TNF, tumor necrosis factor; MAPK, mitogen-activated protein kinase; NaTC-AP, sodium taurocholate-induced acute pancreatitis; SphK1, sphingosine kinase 1; S1P, sphingosine 1-phosphate; TREM-1, triggering receptor expressed on myeloid cells-1; TLR4, Toll-like receptor 4.

#### 3.3.2 Adhesion

Monocyte adhesion to activated endothelial cells is an essential step for the capture, rolling and transmigration of monocytes into target tissues. This process is regulated by a number of different cell surface proteins on both monocytes and endothelial cells. For example, P-selectin glycoprotein ligand 1 (PSGL-1, CD162) modulates the migration, activation, and infiltration of immune cells through binding with E-, P-, or L-selectin (69, 140). Peripheral monocytes from AP patients exhibited increased PSGL-1 expression compared with monocytes from healthy controls (69). Genetic knockout of PSGL-1 in CER-AP mice resulted in less monocyte-endothelial interaction, fewer infiltrating pancreatic monocytes/macrophages and peripheral Ly6C^+^ monocytes and a milder disease course (69). Platelet endothelial cell adhesion molecule 1 (PECAM-1; CD31) and intercellular adhesion molecule 1 (ICAM-1; CD54) are adhesion molecules found on the surface of activated endothelium as well as on some immune cells including monocytes and their expression is upregulated in experimental AP (141). In patients, cellular adhesion molecules in general, but ICAM-1 in particular, have been shown to be expressed in greater amounts in patients experiencing lung injury and respiratory failure in the context of severe acute pancreatitis (67, 139, 142). CD11b, a member of the β2 integrin family of adhesion molecules, is also up-regulated on circulating monocytes in experimental AP rats at 6, 12, 24, and 48 h after disease induction (66), and in AP patients over healthy controls (139). The degree of overexpression in peripheral monocytes was again associated with AP severity and organ dysfunction (107).

#### 3.3.3 Phagocytosis

Phagocytosis by monocytes/macrophages is critical for the clearance of microbial pathogens and apoptotic cells in order to maintain homeostasis; it is a rare event at rest, but increases during inflammation upon stimulation by bacterial products, cellular debris or cytokines (143). The phagocytic activity of monocytes is a measurable entity that may provide further insight about the innate immune response in specific disease conditions (144). Impaired phagocytosis in monocytes is associated with immunosuppression in severe trauma and sepsis (145–147). In AP patients, the phagocytotic (148, 149) and ROS generation capacity (150) of circulating monocytes is impaired and this phagocytosis potential may be a useful predictor for infectious complications. By calculating the area under the receiver-operating-characteristic curve (AUC), a statistical tool routinely used for evaluating the discriminatory ability of continuous markers, it was reported that the admission phagocytosis potential ([ROS generation index value after stimulation-basal value]/basal value) had an AUC of 0.84 with sensitivity and specificity of 76.2% and 83.6%, respectively, for predicting infectious complications in patients with MSAP/SAP (150).

#### 3.3.4 Gaseous signaling

Activated monocytes contribute to gaseous signaling through the generation of carbon monoxide (CO) and nitric oxide (NO). Specifically, CO serves as a gaseous signaling molecule exerting anti-inflammatory effects through regulation of p38 MAPK and IL-10 pathways (151, 152), whereas NO regulates intracellular signaling molecules such as MAPK, JAK, NF-κB, and activating protein-1, mediating the production of pro-inflammatory cytokines and chemokines (153, 154).

CO is a downstream product of heme oxygenase-1 (HO-1) released together with biliverdin and ferritin as a consequence of heme metabolism, all of which contribute to an anti-inflammatory effect (155). Monocytes expressing HO-1 are anti-inflammatory and rapidly recruited to the pancreas dampening the severity of CER-AP and CDE-AP in mice (156). In MAP patients, HO-1 expression of monocytes on admission was higher than healthy controls (156). The level of HO-1 expression was highest on admission and decreased to normal levels on day 3 with clinical improvement (156). The release of CO can be achieved in a controlled way by administration of CO-releasing molecule-2 (CORM-2) without disturbing carboxyhemoglobin levels. CORM-2 suppressed TLR4/MD2 receptor complex activation and TNF-α production by macrophages in pancreas and spleen, attenuating cytokine cascade and organ injury in CER-AP and CDE-AP in mice (157). CORM-2 further inhibited pancreatic CCL2 production and blocked CCL2-triggered CCR2 endocytosis, thus impairing pathological CD11b^+^Ly6C^hi^ monocyte recruitment from the blood to pancreas (94). This axis has also been effectively targeted with therapy using haemin, a HO-1 inducing agent in clinical use for the treatment of thalassemia intermedia. Haemin has been shown to upregulate HO-1 expression in peritoneal and pancreatic macrophages, which reduced severity of CER-AP and CDE-AP in mice (140, 156).

NO is generated *via* the activity of nitric oxide synthase (NOS), which is inducible in inflammatory cells in response to activation (153). Increased expression of iNOS could be detected in peritoneal monocytes/macrophages in NaTC-AP but not CER-AP rats (158), and while low NO concentrations ameliorated microcirculation disturbances, large concentrations contributed to capillary leakage and micro-circulatory failure (159). Clinically, elevated iNOS expression was detected in monocytes from AP patients with systemic inflammatory response syndrome, but no significant changes were observed in those without (160).

## 4 Obesity and lipid modulation of monocytes in AP

Obesity, as defined by a body mass index of 30 kg/m^2^ or more, has reached global pandemic levels (161). Beyond merely an excess in body weight, it is a metabolic disorder associated with excessive lipid storage and chronic, low-grade inflammation (161). The relationship between obesity and AP has been gradually explored using various obesity-related AP animal models. These models include induction of NaTC-AP in high-fat diet (HFD) fed rats (162) and CER-AP (or CER/LPS-AP) in HFD fed, leptin-deficient *ob/ob*, or leptin receptor-defective *db/db* mice (163–165) as well as the administration of IL-12 plus IL-18 in *ob/ob* mice or HFD mice (166, 167). Most recently, our group has shown that administration of alcohol in HFD mice could induce AP with multiorgan injury mediated by adipocyte tissue lipolysis (168). Clinically, studies have linked obesity to an increased incidence and severity of AP (169). Obesity may aggravate AP severity through unsaturated fatty acids *via* lipase mediated lipolysis and various adipokines from adipose tissue (170–172). During AP, pancreatic lipase leaks from the injured pancreas into adipose tissue with increased amount and activity, generating excessive free fatty acids *via* lipolysis, which in turn worsen systemic inflammation and organ failure in AP (173, 174). Genetic knockout or pharmacological inhibition of pancreatic lipase reduced adipose tissue lipolysis, systemic inflammation, organ failure, and mortality of AP in *ob/ob* obese mice (170, 173, 174).

Dyslipidemias are disorders of the plasma lipid profile that are frequently related with obesity and diabetes (175, 176). With the recognition of metabolically healthy obesity and metabolically unhealthy normal weight subjects, the association of obesity and dyslipidemia becomes complex and intriguing (176). Hypertriglyceridemia (HTG) is a highly prevalent form of dyslipidemia with major health concern (175). HTG is the third most common cause of AP globally (177) and is one of the most leading etiologies in Chinese AP cohorts (178–181). Apart from elevated triglyceride levels, HTG-AP is also accompanied with maladjusted lipoprotein metabolism (177). Furthermore, the severity of AP is proportionally increased with escalating triglyceride levels (178, 182, 183).

Adipocytes release lipids through lipase-mediated lipolysis or lipid-filled exosomes, modulating the differentiation and function of monocytes/macrophages (184). Activation and polarization of monocytes/macrophages is affected by lipids *via* direct effects on the physical properties of plasma membranes or indirectly with lipids acting as signaling molecules (185, 186). Distinct lipid species (saturated or unsaturated fatty acids, cholesterols, and lipoproteins), lipid modifications (i.e., oxidation) and dyslipidemias (hypercholesterolemia and hypertriglyceridemia) affect monocyte responses in different ways and relevant studies are summarized in **Supplementary Table 3**. In extremely obese mice, adipose tissue macrophage content is over 50% of the total cell count and most are derived from circulating monocytes (187). Obesity preferentially promotes M1 polarization of monocytes/macrophages in adipose tissue of mice but the phenotypes of adipose tissue macrophages in human are not fully determined (187).

**Figure 3:**
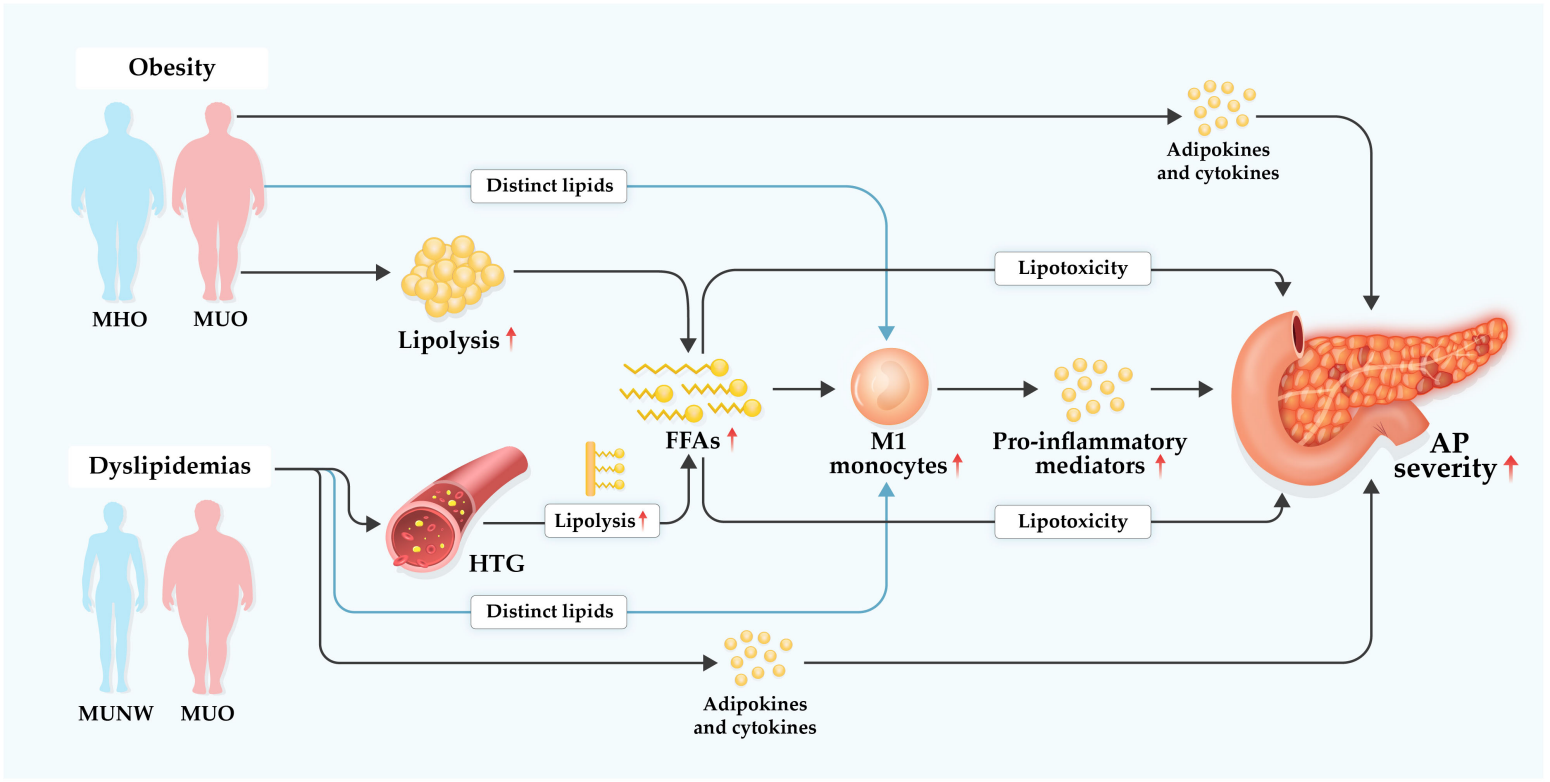
Hypothetical diagram of underlying mechanisms of obesity and dyslipidemias in aggravating acute pancreatitis. Dyslipidemias are prevalent in individuals with metabolically unhealthy obesity (MUO) or metabolically unhealthy normal weight (MUNM), but less prominent or even absent in individuals with metabolically healthy obesity (MHO). Hypertriglyceridemia (HTG), a common form of dyslipidemias, is one of leading etiologies of acute pancreatitis (AP) with increasing global incidence. Obesity, particularly MUO, and dyslipidemias, especially HTG, may exacerbate AP severity in several complex multifactorial ways. Excessive free fatty acids (FFAs) are accumulated *via* elevated lipolysis of visceral adipocyte triglycerides during AP. FFAs possess regulatory effect on monocytes and may promote their M1 polarization which induces increased production of pro-inflammatory mediators, resulting in amplified systemic inflammation. Of note, local and systemic lipotoxicity of FFAs can directly cause tissue damage and multiple organ failure in AP. Besides FFAs, other distinct lipid species may modulate generation and function of monocytes in AP. Furthermore, various adipokines and cytokines with enhanced secretion in MUO and dyslipidemia are involved in boosting systemic inflammatory response that contributes to aggravated severity of AP.

Adipose tissue, particularly areas of fat necrosis, is a vital source of inflammatory mediators promoting the pro-inflammatory M1 polarization of monocytes/macrophages and inhibition of the M2 reparative phenotype, thereby exacerbating systemic inflammation in PDL-AP or NaTC-AP rats (188, 189). It was also observed that oxidized free fatty acids in ascitic fluid interfered with the anti-inflammatory regulation of monocytes/macrophages, aggravating inflammatory responses in NaTC-AP rats (190). As aforementioned, the infiltration of macrophages in adipose tissue is increased in obese mice. After CER/LPS challenge to HFD mice, despite an overall decreased count of adipose tissue macrophages, the proportion of M1 adipose tissue macrophages increased together with enhanced expression of pro-inflammatory cytokines in adipose tissue (165). Consistent with experimental studies, CD14^+^CD86^+^ M1 monocytes increased in circulation in HTG population compared with healthy controls (68). Likewise, HTG-AP patients displayed higher M1 monocyte counts than respective HTG control patients on days 1, 3, and 7 after admission. Most importantly, the level of peripheral M1 monocytes was positively correlated with plasma triglyceride level and Ranson score (a scoring system to assess and prognosticate AP severity) in HTG-AP patients (68). However, there was no obvious difference in peripheral M2 monocytes between HTG-AP patients and HTG control patients (68). Besides, the level of M2 monocytes exhibited no association with either plasma triglyceride level or Ranson score in HTG-AP patients (68). The effects of obesity and dyslipidemias on the recruitment and polarization of monocytes/macrophages in AP are still unclear, however, the mechanism by which obesity and dyslipidemias exacerbate AP severity might, at least in part, be attributed to disturbing monocyte homeostasis (i.e., promoting M1 monocytes polarization). How far this effect is mediated by lipotoxicity of specific lipids/lipid groups warrants further investigation (**Figure 3**).

## 5 Conclusions and perspectives

Due to the difficulties in studying the human pancreas directly, circulating immune cells in general, and monocytes in particular, serve as excellent intermediates for assessing systemic inflammation and immune status. Monocytes play a pivotal role in the initiation and progression of AP, but important questions regarding the precise role of monocytes in the pathogenesis of AP remain. These include:

1. Dynamic changes and roles of monocyte subsets are unclear, as indeed are the degree to which cells can be encouraged into different expression patterns using environmental cues. It is still unknown how the spatially defined signals in the inflamed pancreas serve to reprogram monocyte subsets and determine their fate. Monocytes could extravasate into tissue retaining monocyte phenotypic markers without differentiating into macrophages. Increasing recognition of heterogeneity in monocyte population makes identification of tissue monocytes and deciphering the transition of monocyte subsets into particular tissue macrophages more challenging. Monocytes are remarkably multipotent cells in regulating inflammation but the pathogenetic mechanisms of monocytes in regulating AP severity are not fully investigated. More fundamentally, novel technologies have identified cells based on their distinct transcription patterns, whereby traditionally much simpler phenotypic markers were used. These can greatly compromise comparability between studies. A consensus on relevant monocyte subsets based on multiple different detection technologies, and their lineage, might be developed for better communications between laboratories and future clinical appliance.

2. The finely orchestrated balance between pro- and anti-inflammatory responses is disturbed in more severe forms of AP, which is reflected in imbalances in monocyte function. Obesity and dyslipidemias aggravate AP severity with varying degrees depending on individual factors. Accumulation of free fatty acids generated by elevated lipolysis in patients with metabolically unhealthy obesity and HTG may partially contribute to the exacerbation of AP *via* promoting M1 monocytes polarization, which needs further investigations. Besides free fatty acids, other lipid species are capable of regulating subsets, numbers, and functions of monocytes, but their specific involvement in dynamic changes of monocytes during AP is largely unknown. Future studies, especially those aiming to develop targeted therapy, should design methods to differentiate between causal and associative patterns in monocyte changes with disease in order to guide the development of effective therapy.

3. Immune markers on circulating monocytes provide a potential window for identifying particular inflammation types in diseases. It should be possible to refine the inflammatory signatures of immune cells and develop specific ‘immune signaling fingerprints’ to monitor the inflammatory response and disease progression during AP. These could be used to guide personalized therapeutic strategies using anti-inflammatory, immunomodulatory, immunostimulatory agents alone or in combination for the optimal treatment of individual patients using a personalized medicine approach.

## Author contributions

SL, TL, WH, and QX designed this work and wrote the manuscript. PS and J-WL critically revised the manuscript. QW, RS, and LC had important intellectual input. All authors contributed to the article and approved the submitted version.
