# Supplementary material for: Circulating monocytes in acute pancreatitis
Source: Front Immunol. 2022 Dec 12;13:1062849. doi: 10.3389/fimmu.2022.1062849 (PMC9791207; doi:10.3389/fimmu.2022.1062849)
Supplement: Supplementary file 1 [file Table_1.docx]

Supplementary Material

# SUPPLEMENTARY TABLE 1 | Studies assessing dynamic changes and the mechanisms of monocytes in experimental acute pancreatitis.

| **Studies** | **Models** | **Species (genetic manipulation)** | **Monocytes source** | **Sampling time** | **General findings** |
| --- | --- | --- | --- | --- | --- |
| Goto et al., 1993 (1) | PDL | Wistar rats | Pancreas | On 1, 2, 3, and 4 d after model induction. | The proliferation macrophages reached its peak on 1 d after PDL-AP induction and consisted of heterogeneous subpopulations including monocyte-like cells and resident macrophages. |
| Satoh et al., 1998 (2) | Sodium taurocholate, 5%, 1 mL/kg, *i.d.*  Caerulein, 8 × 50 or 100 μg/kg at 1-h intervals, *i.p.* | Wistar rats | Lavage fluid from peritoneal cavity | At 6, 12, and 18 h after model induction. | (1) The activation of NF-κB was detected in peritoneal macrophages in both models.  (2) The increased expression of iNOS was detected in peritoneal macrophages in NaTC-AP but not in CER-AP;  (3) Ascites from NaTC-AP but CER-AP could induce iNOS production in peritoneal macrophages isolated from normal rats *in vitro.* |
| de Diosm, et al., 2002 (3) | PDL | Wistar rats | Blood | At 1.5, 3, 6, 12, 24, and 48 h after model induction. | (1) The number of monocytes increased up to 6 h after PDL-AP induction.  (2) Monocytes were activated from 6 h with increased CD11b expression and TNF-α production. |
| Liu et al., 2003 (4) | Sodium taurocholate, 3% or 5%, 1 mL/kg, *i.d.* | SD rats | Blood and ascites | At 3 and 6 h after model induction. | Monocytes/macrophages: moderate or strong expression of NF-κB and p38 MAPK in 3% or 5% NaTC-AP, respectively, highest at 6 h, consistent with raised serum levels of TNF-α and IL-6. |
| Dib et al., 2003 (5) | Sodium taurocholate (5%, 0.8 mL/kg), *i.d.* | SD rats | Blood | At 1, 3, 6, and 9 h after model induction. | Hydrogen peroxide generation by circulating monocytes/macrophages began to increase at 1 h in NaTC-AP and further increased thereafter. |
| Zhao et al., 2005 (6) | Sodium taurocholate, 5%, 1 mL/kg, *i.d.* | SD rats | Blood and lung | At 1 and 6 h after model induction. | Cromolyn, a mast cells stabiliser, prevented a decreased expression of PECAM-1 on circulatory monocytes/macrophages and against an increased expression of ICAM-1 and PECAM-1 on pulmonary monocytes/macrophages 6 h after NaTC-AP induction. |
| Shi et al., 2006 (7) | Sodium taurocholate (5%, 0.8 mL/kg), *i.d.* | SD rats | Blood | At 3, 6, and 9 h after model induction. | (1) A marked decrease in the activation of NF-κB in circulating monocytes was seen 6 h after induction of NaTC-AP, opposed to the increased activation of NF-κB in pancreas;  (2) The expressions of IL-6, CINC, and MCP-1 were not significantly changed in circulating monocytes of NaTC-AP at 6 h, while these were increased in pancreas.  (3) An increase in peripheral CD11b^+^ cells was observed at 6 and 9 h after model induction. |
| Rakonczay et al., 2008 (8) | L-ornithine, 30%, 3 g/kg, *i.p.* | Wistar rats | Pancreas | At 2, 4, 6, 9, 12, 18, 24, 36, 48, 72, and 168 h as well as 1 month after model induction. | At 9 h, interstitial monocytic adherence; At 12 h, diffuse moderate infiltrate of monocytes; At 18 h, large numbers of interstitial monocytes; At 36 h, severe but decreased monocytic adherence; At 48 h, diffuse severe infiltrates of monocytes/macrophages; At 72 h, a diffuse severe infiltrate of monocytes/macrophages. |
| Perides et al., 2011 (9) | Caerulein, 12 × 50 μg/kg, at 1-h intervals, *i.p.*  Sodium taurocholate, 50 μL, 37 mM, *i.d.* | FVB/N mice (CD11b-DTR; TNF-α KO) | Bone marrow, blood, and pancreas | At 24 h after model induction. | (1) DT administration to CD11b-DTR mice prevented the increase of pancreatic Ly6C^hi^ monocytes/macrophages and reduced oedema and necrosis in CER-AP and NaTC-AP.  (2) The severity was reversed by adoptive transfer of purified Ly6C^hi^ monocytes from non-DT-treated CD11b-DTR mice or from TNF-α^+/+^ donor mice, but not reversed by the transfer of Ly6C^hi^ monocytes harvested from TNF-α^-/-^ donors. |
| Frossard et al., 2011 (10) | Caerulein, 10 × 50 μg/kg, at 1-h intervals, *i.p.* | DBA/1j × BALB/c Mice/ (CCL2 KO; CCR2 KO; CCR4 KO) | Lung and pancreas | At 1 h after the last caerulein injection. | The deficiency of CCL2, but not CCR2 or CCR4, lowered the number of monocytes/macrophages in pancreas and reduced serum amylase and IL-6 levels, pancreatic and lung injury in CER-AP. |
| Saeki et al., 2012 (11) | Caerulein, 8 × 50 or 100 μg/kg at 1-h intervals, *i.p.*  L-arginine, 2 × 4 g/kg at 1-h interval, *i.p.* | C57BL/6 mice (CCL2 KO; SOCS3c KO) | Bone marrow, spleen, blood, and pancreas | At 1 h after the last caerulein injection and 72 h after the first L-arginine injection. | (1) Almost all types of immune cells except for the CD11b^high^CD11c^-^ population residing in pancreas under steady state condition while CD11b^high^ CD11c^-^ Gr-1^low/high^ cells were detected after caerulein stimulation.  (2) *Ccl2^-/-^* mice developed less severe CER-AP, with less infiltration of CD11b^high^CD11c^-^Gr-1^low^ macrophages.  (3) Parabiosis and bone marrow analyses suggested that CD11b^high^CD11c^-^Gr-1^low^ macrophages had moved from bone marrow.  (4) *Socs3c^-/-^* mice developed less severe CER-AP with unchanged number of CD11b^high^CD11c^-^Gr-1^low^ macrophages. But the up-regulation of CD80 and CD86 as well as the production of TNF-α after culture of those CD11b^high^CD11c^-^Gr-1^low^ macrophages were impaired.  (5) CCL2-mediated migration of macrophages was also involved in development of ARG-AP. |
| Guo et al., 2015 (12) | L-arginine, 1 mL, 0.4 g/kg, injected into 5 different locations of pancreas | SD rats | Pancreas | At 4, 12, 24, 48, 72, and 96 h after model induction. | From 4 to 12 h, a small infiltration of monocytes; At 24 h, more evident infiltration of monocytes. |
| Schmidt et al., 2015 (13) | Sodium taurocholate, 4%, 2 mL/kg, *i.d.* | Balb/c mice | Spleen, blood, and liver | At 6, 12, 24, and 48 h after model induction. | The number and the expression of activation markers (CD121b/IL1R2 and MHCII) of monocytes/macrophages increased in spleen, blood, and liver during NaTC-AP. |
| Yu et al., 2016 (14) | PDL | Wistar and Lewis rats | Pancreas | On 1, 2, 3, and 4 d after model induction. | In the PDL-AP, pancreatic infiltrating macrophages mainly divided into two distinct subpopulations:  (1) Monocyte-derived macrophages with either the M2 phenotype (CD68^+^CD163^+^CD206^+^arginase 1^+^) in the inter-lobular area.  (2) Non-M2 phenotype CD68^+^CD163^−^CD206^−^arginase 1^−^) in the inter-acinar area which partly included the NOS2^+^ M1 phenotype. |
| Wu et al., 2020 (15) | Caerulein, 10 × 100 μg/kg, at 1-h intervals, *i.p.* | C57BL/6j mice (IL4Ra KO; CCR2 KO); B6.SJL  × C57BL/6j mice (CD45.1/CD45.2); Balb/c mice | Pancreas | On 1, 3, 5, and 7 d after model induction. | (1) M2-like macrophages, mainly derived from CCR2^+^ monocytes/macrophages recruited at early stage, dominated during pancreas repair/regeneration.  (2) Depletion of macrophages at early or late regenerative stage dramatically blocked the ADM or delayed inflammation resolution, respectively.  (3) Blocking macrophage PI3K-AKT activation during ADM triggered reflux of inflammatory cells (elevated neutrophils, inflammatory monocytes and immature macrophages) and tissue damage.  (4) *Il4ra*^-/-^ mice had decreased M2-like macrophages and more ADM structure.  (5) PGE2 augmented IL4Ra signalling to enhance M2 activation of pancreatic macrophages. |
| Zhang et al., 2020 (16) | Caerulein, 7 × 200 μg/kg, at 1-h intervals, *i.p.* + LPS, 10 mg/kg, single injection, *i.p.* | Mice (PSGL-1 KO) | Blood and pancreas | At 24 h after model induction. | *Psgl-1*^-/-^ mice had reduced accounts of peripheral monocyte and infiltration of pancreatic monocytes/macrophages, resulting in alleviated acinar damage and inflammatory response (less IL-1β and IL-6 expression, lower serum amylase) of CER/LPS-AP. |
| Manohar et al., 2021 (17) | Caerulein, 7 × 50 μg/kg, at 1-h intervals, *i.p.*  CDE diet | Balb/c mice | Blood and pancreas | At 12, 24, 48, and 168 h after CER-AP and at 0, 24, 48, and 72 h after CDE-AP. | (1) Ly6C^+^ inflammatory monocytes were amongst top changed immune cells in the pancreas during CER-AP and its recovery as well as CDE-AP.  (2) Newly identified monocyte and CD206^+^ macrophage subsets had significantly altered surface (CD44, CD54, CD115, CD140a, CD196, and podoplanin) and functional markers (interferon-γ, IL-4, IL-22, LAP-TGF-β, TNF-α, T-bet, and RoRγt) that were associated with CER-AP recovery and CDE-AP. |

Abbreviations: PDL, pancreatic duct ligation; PDL-AP, pancreatic duct ligation-induced acute pancreatitis; *i.d.,* intraductally; *i.p.*, intraperitoneally; NF-κB, nuclear transcription factor-κB; iNOS, inducible nitric oxide synthase; NaTC-AP, sodium taurocholate-induced acute pancreatitis; CER-AP, caerulein-induced acute pancreatitis; TNF-α, tumour necrosis factor-alpha; SD, Sprague-Dawley; MAPK, mitogen-activated protein kinase; IL, interleukin; PECAM-1, platelet endothelial cell adhesion molecule-1; ICAM-1, platelet endothelial cell adhesion molecule-1; CINC, cytokine-induced neutrophil chemoattractant; MCP-1, monocyte chemoattractant protein-1; DT, diphtheria toxin; DTR, diphtheria toxin receptor; SOCS3, suppressor of cytokine signalling 3; _(c)_KO, (conditional) knockout; ARG-AP, L-arginine-induced acute pancreatitis; MHCII, major histocompatibility complex class II; LPS, lipopolysaccharide; NOS2, nitric oxide synthase 2; PI3K, phosphatidylinositol 3-kinase; ADM, acinar-ductal metaplasia; PGE2, prostaglandin E2; PSGL-1, P-selectin glycoprotein ligand 1; CER/LPS-AP, caerulein plus lipopolysaccharide-induced acute pancreatitis; CDE, choline-deficient DL-ethionine; CDE-AP, choline-deficient DL-ethionine diet-induced acute pancreatitis; LAP, latency associated peptide; TGF-β, transforming growth factor-beta; T-bet, T-box expressed in T cells; RoRγt, retinoid-related orphan receptor-gammat.

# SUPPLEMENTARY TABLE 2 | Studies assessing dynamic changes and the mechanisms of monocytes in clinical acute pancreatitis.

| **Studies** | **Population** | **Severity definition** | **Recruit time** | **Origin of monocytes** | **Sampling time** | **General findings** |
| --- | --- | --- | --- | --- | --- | --- |
| Larvin et al., 1993 (18) | HC (n = 8)  MAP (n = 20) SAP (n = 9) | Major OF or pancreatic collections | NA | Blood | NA | (1) Monocyte phagocytosis capacity: SAP < MAP or HC.  (2) Monocyte phagocytosis capacity and plasma trypsin half-life were inversely correlated. |
| Liras et al., 1996 (19) | HC (n = 12) MAP (n = 14) SAP (n = 7) | Imrie classification | < 12 h | Blood | At 24 h, 48 h, 72 h, and on 5 d | Monocyte phagocytosis capacity during 24-72 h: SAP < MAP. |
| Mckay et al., 1996 (20) | AP without biliary sepsis  (n = 26) | Admission APACHE II score > 5 | NA | Blood | At 36 h (range 12-102) after pain onset | Cultured monocytes isolated from peripheral blood of AP with systemic complications after LPS stimulation for 24 h secreted higher TNF-α, IL-6, and IL-8 but not IL-1β *in vitro* compared with those without systemic complications. |
| Salomone et al., 1996 (21) | MAP (n = 16)  SAP (n = 5) | OAC | < 24 h | Blood | On 1-2 d, 3-4 d, and 5-6 d | During the observational period, the percentage of monocytes to peripheral WBCs was positively correlated with serum soluble IL-2R levels and the percentage of lymphocytes to WBCs. |
| Richter et al., 1999 (22) | MAP (n = 12) SAP (n = 25) | OAC | < 24 h | Blood | On 1, 3, 5, 7, 9, and 11 d | (1) Monocyte HLA-DR expression was depressed in AP on 1-5 d but recovered back to normal on 7 d in MAP.  (2) Monocyte HLA-DR expression was reduced in non-survivors from 7 d onwards compared with survivors of SAP. |
| Gotzinger et al., 2000 (23) | HC (n = 7)  MAP (n = 5)  SAP (n = 9) | OAC | NA | Blood | NA | (1) Monocyte HLA-DR and CD14 expressions: SAP < MAP or HC.  (2) Monocyte HLA-DR and CD14 expressions were not affected by operation procedures. |
| Bhatnagar et al., 2001 (24) | Autopsy controls  (n = 12)  AP (n = 34) | NA | NA | Pancreas | During necrosectomy | Higher LPS-induced increase of IL-6 and IL-12 levels in monocytes isolated from pancreas of AP compared with those of autopsy cases. |
| Bhatnagar et al., 2001 (25) | HC (n = 20)  Autopsy controls (n = 15)  AP (n = 34) | NA | NA | Blood and pancreas | NA | (1) Increased expression of activation markers (CD69, CD25) and adhesion molecules (CD54/ICAM-1, CD11a, CD11b) and decreased expression of HLA-DR were observed in mononuclear cells from and blood (vs. HC) and pancreas (vs. autopsy) of AP.  (2) TNF-α-producing monocytes were elevated in blood and pancreas of AP compared with respective controls. |
| Kylanpaa-Back et al., 2001 (26) | HC (n = 30)  MAP (n = 58)  SAP (n = 31) | OAC | < 72 h | Blood | On admission, at 12, 24, 36, and 48 h | (1) Monocyte HLA-DR expression: 1 d or 2 d < on admission; SAP with OD < SAP without OD < MAP.  (2) Monocyte CD11b expression was persistently high in SAP with OD.  (3) Monocyte CD14 and L-selectin expressions were not different between MAP, SAP without or with OD. |
| Satoh et al., 2002 (27) | MAP (n = 36)  SAP (n = 28) | OAC | < 72 h | Blood | On admission, 7d, and 14 d after pain onset | (1) Monocytic HLA-DR expression on admission: SAP (84%) < MAP (98.5%); AP with sepsis (23.5%) < AP without sepsis (96%); negatively correlated with Ranson (r = -0.57) and the APACHE II (r = -0.51) scores.  (2) Predicting sepsis: monocytic HLA-DR expression at a cut-off value ≤ 60% had sensitivity of 100%, 100%, and 100%, and specificity of 91.3%, 93.2%, and 98.2% on admission, 7 d, and 14 d, respectively, better than Ranson and APACHE II scores. |
| Mentula et al., 2003 (28) | MAP (n = 194)  SAP (n = 116) | OAC | < 72 h | Blood | On admission | (1) Monocyte HLA-DR expression: SAP with OD (62% [49-76%]; 20 RFU [13-49]) < SAP without OD (84% [66-91%]; 65 RFU [34-115] < MAP (90% [80-95%]; 90 RFU [53-143]).  (2) Predicting OD: proportion of HLA-DR^+^ monocytes at a cut-off value ≤ 78% had sensitivity of 83% and specificity of 72%; HLA-DR density at a cut-off value ≤ 33 RFU had sensitivity of 69% and specificity of 84%. |
| Mentula et al., 2004 (29) | MAP (n = 27)  SAP (n = 47) | OAC | < 72 h | Blood | On admission, 1 d, 2 d, 3 d, 7 d, 14 d, and 21 d | (1) The proportion of HLA-DR^+^ monocytes on 1-3 d, 7 d, and 14 d (SAP with OF < SAP without OF < MAP).  (2) The proportion of HLA-DR^+^ monocytes was comparable in MAP between on admission, 1 d, 2 d, and 3 d, while downregulated and was lowest on 2 d in SAP which negatively correlated with the peak concentrations of plasma IL-1ra, IL-6, and IL-10 levels during on admission, 1 d, and 2 d.  (3) Monocyte HLA-DR expression: SAP with infection < SAP without infection on 14 d (32% [23-61%] vs. 65% [40-80%]) and 21 d (49% [36-69%] vs. 83% [62-96%]).  (4) Predicting SAP or OF: AUC for HLA-DR^+^ monocytes proportion on admission was 0.769 and 0.652, respectively.  (5) Monocyte HLA-DR expression did not differ between survivors (56% [47-62%]) and non-survivors (61% [52-64%]) of SAP with OF on 1 d. |
| Yu et al., 2004 (30) | HC (n = 10)  MAP (n = 25)  SAP (n = 49) | OAC | < 48 h | Blood | On 1, 2, 3, 7, 10 and 14 d | (1) Proportion of HLA-DR^+^ monocytes: AP (65.6 ± 20.4%) < HC (94.4 ± 4.2%). The proportion of HLA-DR^+^ monocytes was lowest on 3 d and inversely correlated with APACHE II and MODS scores as well as serum IL-6 and CRP levels.  (2) Predicting septic complications: on 7, 10, and 14 d, the proportion of HLA-DR^+^ monocytes at the cut-off value ≤ 40% had sensitivity and specificity of 73% and 94%, 82% and 98%, 100% and 100%, respectively.  (3) During the entire observation period, the HLA-DR^+^ monocytes were lower in non-survivors compared with survivors of SAP. |
| Rahman et al., 2004 (31) | HC (n = 263);  AP (n = 117):  MAP (n = 83)  SAP (n = 34) | OAC | < 24 h | Blood | At 24 h and 72 h after pain onset | (1) Plasma soluble CD14 levels (in ng/mL) were higher in SAP than MAP (24 h: 66.6 [25–216] vs. 50.7 [24–103]; 72 h: 72.2 [32–340] vs. 49.7 [16–186]); those in MAP were comparable to HC (52.5 [30-78]); significantly correlated with respective APS (24 h: r = 0.43; 72 h: r = 0.60), PBMC CD14^+^CD16^+^ receptor densities (24 h: r = 0.43) and CRP (24 h: r = 0.3; 72 h: r = 0.3).  (2) PBMC CD14^++^, CD14^+^CD16^+^ and CD16^++^ receptor densities were all increased in SAP compared with MAP at 24 h (MAP vs. SAP, CD14^++^ [13% vs. 20.5%], CD14^+^16^+^ [4.7% vs. 7.3%], CD16^++^ [15.1% vs. 19.3%]).  (3) The CD14 genotype prevalence in AP was similar to HC. |
| Kylanpaa et al., 2005 (32) | HC (n = 28)  SAP (n = 28) | OAC | NA | Blood | On admission, 1-4, 7, 14, and 21 d | (1) Proportion of HLA-DR^+^ monocytes: AP (45% [18-73%]) < HC (98% [86-100%]).  (2) Lower proportion of HLA-DR^+^ monocytes and reduced LPS-stimulated TNF-α of monocytes were observed in AP compared with HC, which were inhibited by GM-CSF, IFN-γ or their combination.  (3) The proportion of HLA-DR^+^ monocytes was correlated with blood TNF-α levels after LPS stimulation (r = 0.56). |
| Lindstrom et al., 2006 (33) | SAP (n = 31) | OAC | NA | Blood | On 1, 3, 5, 7, and 10 d | The proportion of HLA-DR^+^ monocytes did not differ between SAP with ((46% [8–82%]) or without MODS (44% [11–84%]) but positively correlated with plasma protein C and activated protein C levels during the stay in ICU. |
| Ho et al., 2006 (34) | SAP (n = 25) | OAC | < 72 h | Blood | At < 24 h of admission and on 4, 7, 10, and 14 d | (1) Predicting late mortality: on 10 d, monocyte HLA-DR expression at the cut-off value ≤ 52.3% had AUC of 0.944 with sensitivity of 94.4% and specificity of 85.7%, comparable to Ranson and APACHE II scores.  (2) Predicting septic complications: on 10 d, monocyte HLA-DR expression at the cut-off value ≤ 58.4% had AUC of 0.926 with sensitivity of 76.5% and specificity of 100%, comparable to Ranson and APACHE II scores.  (3) Monocytes from SAP had lower HLA-DR expression after LPS stimulation than HC. |
| Li et al., 2007 (35) | HC (n = 20)  MAP (n = 30)  SAP (n = 6) | OAC | < 24 h | Blood | On admission, 1, and 3 d | (1) Monocyte TLR4 expression increased on 1 d in MAP compared with HC and reduced thereafter almost back to normal on 7 d, coincident with changes of serum TNF-α and IL-6 levels.  (2) Monocyte TLR4 expression and serum cytokines had irregular changes in SAP during the study period. |
| Mentula et al., 2007 (36) | AP (n = 117) | OAC | < 72 h | Blood | On admission | (1) Monocytic HLA-DR expression on admission: AP with OF (60% [50-75%]) < AP without OF (86% [77-95%]).  (2) Monocytic HLA-DR expression of AP without OF on admission negatively correlated with BMI (r = -0.34) but the correlation was not significant in AP with OF. |
| Dabrowski et al., 2008 (37) | HC (n = 15)  MAP (n = 20)  SAP (n = 15) | OAC | < 48 h | Blood | On 1, 2, 3, 5, 10, and 30 d | (1) Total peripheral monocyte counts: MAP > HC on 1 and 2 d; SAP > HC on 1, 2, 3, 5, and 10 d; SAP > MAP on 1, 3, 5, and 10 d.  (2) Percentage of monocytes in WBCs: SAP (2 d: 8.4 ± 1.0%, 3 d: 8.3 ± 0.6%, 5 d: 9.0 ± 0.8%) > HC (6.5 ± 1.5%).  (3) Proportion of HLA-DR^+^ CD14^+^ monocytes: SAP (1 d: 89.7 ± 3.6%, 5 d: 92.9 ± 1.2, 10 d: 96.7 ± 1.5%), MAP (1 d: 91.2 ± 7.3%, 5 d: 83 ± 14.9, D10: 89.8 ± 3.5), HC (80.9 ± 5.7%); 5 and 10 d: SAP > MAP > HC.  (4) A marked increase of monocytes was observed in SAP concurrent with increased expression of activation markers (HLA-DR, CD54/ICAM-1, CD69, and CD25) and apoptosis marker (CD95/FasR) compared with HC. |
| Ferat-Osorio et al., 2009 (38) | HC (n = 50)  MAP (n = 18)  SAP (n = 11) | OAC | < 72 h | Blood | At < 24 h of admission, on 1 and 3 d | (1) Monocytic TREM-1 expression on admission, 1, and 3 d was higher in AP than HC but was not associated with severity, mortality, or infection, while serum soluble TREM-1 was higher in non-survivors than survivors.  (2) Monocytic HLA-DR expression: SAP < MAP on 3 d; AP with infection < AP without infection on 3 d; on-survivors < survivors on 1 and 3 d. |
| Oiva et al., 2010 (39) | HC (n = 13)  SAP (n = 13) | OAC | NA | Blood | 4-39 d after admission ∗ | (1) Proportion of HLA-DR^+^ monocytes: SAP (55.0 ± 4.1%) < HC (93.1 ± 3.4%).  (2) The pNF-κB expression in monocytes treated with TNF, LPS, MDP, and whole bacteria (*E. coli*, *S. aureus* or *S. epidermidis*) was lower in AP than in HC.  (3) Monocytes from AP exhibited lower IL-6-induced pSTAT1 and pSTAT3 expression, reduced PMA- and calcium ionophore-induced pERK1/2 levels and impaired MCP-1 induced transmigration through endothelium than HC. |
| Ho et al., 2011 (40) | HC (n = 24)  MAP (n = 25)  SAP (n = 29) | OAC | < 72 h | Blood | On 1, 7, and 14 d | (1) Monocytic HLA-DR expression: SAP (42.3 ± 11.5%) < MAP (86.9 ± 14.6%) or HC (93.9% ± 7.4%) on D1; SAP < HC at 7 and 14 d.  (2) TNF-α increased IL-10 secretion and downregulated HLA-DR expression of sorted normal monocytes in vitro; anti-IL-10 monoclonal antibody did not inhibit the suppression of HLA-DR expression of monocytes induced by TNF-α. |
| Habtezion et al., 2011 (41) | HC (n = 7)  MAP (n = 18) | OAC | NA | Blood | On 1 and 3 d | (1) Monocytic HO-1 expression was higher in AP than HC on 1 d and reduced upon recovery.  (2) Monocytes from AP on 1 d exhibited higher panhematin-induced HO-1 expression than HC. |
| Li et al., 2012 (42) | HC (n = 12)  SAP (n = 22) | OAC | NA | Blood | On 1, 3, 5, 7, and 14 d after pain onset | (1) Peripheral monocyte counts: SAP < HC on 1 and 3 d; SAP comparable to HC on 7 d.  (2) Percentage of monocyte HLA-DR expression: SAP (31.1 ± 13.6%) < HC (91.2 ± 5.1%) at early stage and it increased to 73.4 ± 13.1% in SAP in the restoration stage.  (3) HLA-DR expression on monocytes negatively correlated with APACHE II scores (r = -0.489).  (3) Expression of SphK1 on peripheral monocytes: SAP (45.9 ± 7.5) > HC (28.9 ± 4.0) in the early stage; it reduced in SAP in the restoration stage.  (4) Expression of SphK1 on peripheral monocytes correlated positively with APACHE II scores in SAP (r = 0.545). |
| Oiva et al., 2013 (43) | HC (n = 13)  SAP (n = 13) | OAC | < 72 h | Blood | NA ∗ | Proportion of HLA-DR^+^ monocytes: SAP (55.0% ± 4.1%) < HC (93.1% ± 3.4%). |
| Li et al., 2013 (44) | SAP (n = 77) | OAC | < 24 h | Blood | On 1, 3, 7, and 14 d | (1) Proportion of HLA-DR^+^ monocytes in SAP, 1 d: 79.1 ± 19.7% > 3 d: 68.2 ± 19.5% > 7 d: 57.5 ± 18.9% or 14 d: 57 ± 18.8%.  (2) Proportion of HLA-DR^+^ monocytes negatively correlated with urinary lactulose/mannitol ratio (r = 0.752) and D-lactate levels (r = 0.759). |
| Qin et al., 2013 (45) | HC (n = 28)  MAP (n = 28)  SAP (n = 20) | OAC | NA | Blood | At < 48 h | (1) Monocyte HLA-DR expression: SAP with sepsis (24.6 ± 4.9%) < SAP without sepsis (48.8 ± 4.7%) < MAP (68.8 ± 9%) < HC (87.4 ± 9.7%).  (2) Monocyte HLA-DR expression negatively correlated with Fas mRNA expression of blood (r = -0.884). |
| Lin et al., 2013 (46) | SAP (n = 40) | OAC | < 48 h | Blood | On 1, 3, 5, and 7 d | (1) Monocytic HLA-DR expression: SAP with infection < SAP without infection on 1-7 d.  (2) Monocytic HLA-DR expression on admission negatively correlated with APACHE II score (r = -0.790) and serum CRP (r = -0.642).  (3) Predicting secondary infection: monocytic HLA-DR expression at the cut-off value ≤ 35.8% had AUC of 0.837 (95% CI: 0.685–0.989) with sensitivity of 81.8% and specificity of 82.8%. |
| Zhang et al., 2017 (47) | HC (n = 13)  MAP (n = 24) | RAC | NA | Blood | At < 72 h after pain onset | (1) Numbers of CD14^+^CD163^-^, CD14^+^CD163^-^MAC387^+^, CD14^+^CD163^-^IL-12^+^ M1 monocytes and CD14^+^CD163^+^CD115^+^(CD206^+^) M2 monocytes increased, while CD14^+^CD163^+^IL-10^+^ M2 monocytes decreased in MAP compared with HC.  (2) In MAP patients, CD14^+^CD163^-^, CD14^+^CD163^-^MAC387^+^ M1 monocytes counts and CD14^+^CD163^+^CD115^+^ M2 monocytes counts positively correlated with plasma CRP levels (r = 0.5009, 0.5079, and 0.4565 respectively).  (3) The counts of CD14^+^CD163^+^CD115^+^ M2 monocytes positively correlated with APACHE II score (r = 0.4581) and plasma IL-10 (r = 0.4178) in MAP. |
| Li et al., 2017 (48) | HC (n = 32)  MAP (n = 197)  MSAP (n = 76) SAP (n = 86) | RAC | NA | NA | NA | Predicting mortality: LMR at the cut-off value ≤ 1.40 had AUC of 0.710 (95% CI: 0.660–0.757) with sensitivity of 77.4% and specificity of 54%. |
| Pan et al., 2017 (49) | HC (n = 32)  MAP (n = 3)  MSAP (n = 28)  SAP (n = 32) | RAC | < 24 h | Blood | On 1 and 3 d after pain onset | (1) Increased PD-L1-expressing CD14^+^ monocytes and reduced HLA-DR expressing CD14^+^ monocytes in AP especially with infectious complications on 1 and 3 d compared with HC.  (2) The percentage of PD-L1-expressing CD14^+^ monocytes negatively correlated with lymphocyte counts on 1 d (r = -0.302) and positively correlated with plasma IL-10 levels on 1 d (r = 0.296) and 3 d (r = 0.459).  (3) Percentage of HLA-DR-expressing CD14^+^ monocytes on 1 d (OR 0.923, 95% CI: 0.869-0.981) and PD-L1-expressing CD14^+^ monocytes on 1 d (OR 1.098, 95% CI: 1.005-1.200) were independent predictors of infectious complications.  (4) Predicting infectious complications: the AUC of percentage of HLA-DR-expressing CD14^+^ monocytes, PD-L1-expressing CD14^+^ monocytes and the combination of those with APACHE II score on admission were 0.652 (95% CI: 0.506-0.798), 0.708 (95% CI: 0.573-0.842), and 0.904 (95% CI: 0.816–0.991) respectively. |
| Chen et al., 2017 (50) | HC (n = 21)  MAP (n = 3) MSAP (n = 24) SAP (n = 29) | RAC | < 48 h | Blood | NA | (1) Plasma soluble PD-L1 negatively correlated with monocyte HLA-DR expression in AP (r = -0.311).  (2) Predicting infectious complications: the odds ratio of monocyte HLA-DR expression was 0.950 (95% CI: 0.911-0.991). |
| Sharma et al., 2017 (51) | HC (n = 10)  MAP (n = 14) MSAP (n = 10)  SAP (n = 18) | RAC | < 72 h | Blood | On admission and 2 weeks after pain onset | The relative risk (95% CI) of developing primary IPN with persistent HLA-DR down-regulation of PBMCs till the second week (11.3 [1.6–82.4]) in AP. |
| Sendler et al., 2018 (52) | ANP (n = 15)  CP (n = 13) | NA | NA | Pancreas | During endoscopic necrosectomy | (1) CD68^+^ M1 and CD206^+^ M2 macrophages were detected in pancreatic tissue of CP and ANP.  (2) Co-localisation of trypsinogen and CD68 was observed, whereas co-localisation of CD206 and trypsinogen was excluded. These findings suggested that CD68+ macrophages could phagocytose acinar cell components, including zymogen-containing vesicles, in pancreas of human necrotic pancreatic tissues. |
| Kuuliala et al., 2018 (53) | HC (n = 28)  MAP (n = 9)  MSAP (n = 6)  SAP (n = 3) | RAC | < 72 h | Blood | At < 48 h | (1) Monocytic pSTAT1 expression induced by IL-6: SAP (2.2% [1.2-3.8%]) < MSAP (4.6% [4.2-6.7%]) < MAP (9.1% [2.7-36.1%]) < HC (52.2% [39.3-60.0%]); AP (670 RFU [615-801]) < HC (751 RFU [703-873])  (2) Constitutive pSTAT3 of monocytes: AP (60.6% [25.7-82.5%]) > HC (< 5%)  (3) Monocyte pSTAT3 expression induced by IL-6: AP (95.3% [74.8-97.6%]) < HC (98.3% [95.8-99.1%]); SAP (1227 RFU [557-1632]) < MSAP (1555 RFU [813-3408]) < MAP (2258 RFU [1565-4349]) < HC (2911 RFU [1286-3869]); AP with OD (1065 RFU [850-1848]) < AP without OD (2055 RFU [1692-3456]). |
| Zhang et al., 2018 (54) | HC (n = 13)  MAP (n = 15)  SAP (n = 21) | RAC | < 72 h | Blood | At 24-48 h after admission, before and after 9–11 weeks of treatment | (1) The numbers of CD14^+^CD163^−^, CD14^+^CD163^−^MAC387^+^, CD14^+^CD163^−^IL-12^+^ M1 monocytes, and CD14^+^CD163^+^CD115^+^, CD14^+^CD163^+^CD204^+^, CD14^+^CD163^+^CD206^+^ and CD14^+^CD163^+^IL-10^+^ M2 monocytes were augmented in SAP compared with MAP or HC.  (2) The number of CD14^+^CD163^−^ (r = 0.6606), CD14^+^CD163^−^MAC387^+^ (0.4651) M1 monocytes positively correlated with plasma CRP level, while the number of CD14^+^CD163^+^CD204^+^ (r = 0.7101) and CD14^+^CD163^+^IL-10^+^ (r = 0.5823) M2 monocytes positively correlated with APACHE II score; The CD14^+^CD163^+^CD115^+^ M2 subset correlated positively with plasma CRP level (r = 0.469) and APACHE II score (r = 0.7434).  (3) Treatment of SAP significantly reduced numbers of CD14^+^CD163^−^, CD14^+^CD163^−^MAC387^+^, CD14^+^CD163^−^IL-12^+^ M1 monocytes and CD14^+^CD163^+^CD115^+^, CD14^+^CD163^+^CD204+, and CD14^+^CD163^+^IL-10^+^ M2 monocytes. |
| Yu et al., 2018 (55) | SAP (n = 24) | RAC | < 72 h | Blood | On 1, 7, 14, and 28 d | Percentage of HLA-DR+ monocytes: AP with MDR infection (7.1 ± 2.2%) < AP with non-MDR infection (18.1 ± 14.1%) < AP without infection (35.8 ± 8.32%) on 1 d; AP with MDR infection < normal levels at all time points; AP with non-MDR infection increased after 1 d, comparable to AP without infection on 28 d; AP without infection remained at high levels throughout the study period. |
| Djordjevic et al., 2018 (56) | AP with sepsis  (n = 67) | NA | NA | Blood | On admission | (1) Predicting mortality: the AUC of combination of MPV/PC, NLR, MLR, and PLR in a Composite Bioscore at the cut-off value ≥ 2 was 0.874 (95% CI: 0.791–0.956) with the sensitivity of 72.5% and specificity of 79.5%.  (2) MLR negatively correlated with MPV/PV in non-survivors (r = −0.418). |
| Qiu et al., 2018 (57) | HC (n = 21)  MAP (n = 46)  SAP (n = 17) | RAC | < 24 h | Blood | On admission, 3 and 7 d | (1) CD19^+^CD24^hi^CD27^hi^ cells from AP suppressed the TNF-α productions of CD14^+^ monocytes after co-culture in vitro.  (2) The frequencies and numbers of CD14^+^HLA-DR^low/-^ cells on admission were significantly increased in AP especially SAP patients compared with HC.  (3) Predicting SAP: the AUC of CD14^+^HLA-DR^low/-^ cells counts on admission was 0.745 (95% CI: 0.684–0.897). |
| Kolber et al., 2018 (58) | MAP (n = 29) MSAP (n = 58)  SAP (n = 8) | RAC | < 24 h | Blood | On 1, 2, and 3 d after pain onset | LMR: AP with OF < AP without OF on 1 d; ns. on 2 and 3 d. |
| Liu et al., 2019 (59) | HC (n = 10)  MAP (n = 12) MSAP (n = 1)  SAP (n = 2) | RAC | NA | Blood | On admission | Predicting persistent OF in all patients: the AUC of LMR at the cut-off value ≤ 1.29 was 0.680 (0.622-0.735) with the sensitivity of 62.50% and specificity of 68.63%, comparable with BISAP score. |
| Zhang et al., 2019(60) | HC (n = 9)  MAP (n = 27) MSAP (n = 14)  SAP (n = 9) | RAC | < 48 h | Blood | On 1-2 and 6-8 d | (1) CD14^hi^CD16^-^ monocytes: SAP > MSAP > MAP > HC on 1-2 d, MSAP decreased within 1 week after AP onset while the levels in SAP remained high.  (2) Percentage of CD14^hi^CD16^-^ monocytes on 1-2 d positively correlated with plasma G-CSF (r = 0.308), IL-6 (r = 0.344), IL-10 (r = 0.500), IL-1RA (r = 0.382), and IL-15 (r = 0.331).  (2) HLA-DR expression on CD14^hi^CD16^-^ monocytes: SAP, MSAP < MAP, MSAP increased within 1 week after AP onset while SAP remained at low levels.  (3) HLA-DR expression level on circulating CD14^hi^CD16^-^ monocytes was negatively correlated with the level of CRP as well as plasma IL-6 (r = -0.451), IL-10 (-0.523), IL-8 (-0.368), IL-15 (-0.343), and IL-1RA1 (r = -0.323).  (4) Differentiating MAP from MSAP/SAP: Proportion of CD14^hi^CD16^-^ monocytes, their HLA-DR levels and their combination at the cut-off value ≤ 5.6% and 2274 RFU respectively had AUC of 0.781 (0.640–0.922), 0.805 (0.685–0.925) and 0.862 (0.762–0.961) respectively with the sensitivity of 82.6%, 70.4%, and specificity of 74.1%, 82.6%.  (5) Predicting SAP among MSAP/SAP patients: percentage of CD14^hi^CD16^-^ monocytes and HLA-DR levels at the cut-off value ≤ 16.1% and 1094.5 RFU had AUC of 0.730 (0.520–0.940) and 0.690 (0.441–0.940) respectively with the sensitivity of 66.7%, 85.7% and specificity of 71.4%, 55.6% respectively. |
| Zheng et al., 2019 (61) | HC (n = 20)  HTG (n = 20) ABP (n = 34, MAP/SAP = 22/12)  HTG-AP (n = 16, MAP/SAP = 9/7) | RAC | NA | Blood | On 1, 3, and 7 d | (1) Total monocytes on 1, 3, and 7 d: ABP > HC, HTG-AP > HTG, SAP > MAP.  (2) CD14^+^CD86^+^ M1 monocytes: ABP > HC, HTG-AP > HTG, SAP > MAP, HTG-AP comparable to ABP, severe HTG-AP > severe ABP on 1, 3, and 7 d; MAP, highest on 1 d and decreased until 7 d; SAP, increased to highest on 3 d and slightly decreased on 7 d.  (3) CD14^+^CD206^+^ M2 monocytes: ABP comparable to HC; HTG-AP comparable to HTG; 3 d, HTG-AP < ABP; 3 and 7 d, severe HTG-AP < severe ABP; 3 d, MAP > SAP; MAP increased from 1 to 7 d; SAP, decreased to lowest on 3 d and recovered on 7 d comparable to values on 1 d.  (4) M1 monocyte correlation with triglyceride levels on 1 d: percentage of M1 monocytes, HTG (r = 0.637); number of M1 monocytes, HTG-AP (r = 0.515)  (5) M1 monocyte correlation with Ranson score on 1 d: number and percentage of M1 monocytes, HTG-AP (r = 0.5165 and 0.6988 respectively) |
| Waller et al., 2019 (62) | HC (n = 9)  MAP (n = 11) | RAC | < 24 h | Blood | At < 24 h | (1) CD14^++^CD16^+^ intermediate monocytes: MAP (2.4%) < HC (3.1%).  (2) LPS-induced pNF-kBp65 in monocytes: MAP < HC.  (3) Stimulating PBMCs from HC with TLR agonists LPS, flagellin or poly I:C in vitro resulted in loss of intermediate monocytes via shedding of CD14 and CD16, which was reversed by ADAM17 inhibitor, TMI005.  (3) Culturing PBMCs from HC leaded to expansion of intermediate monocytes, which did not occur with the addition of LPS to the culture medium. Cultured intermediate monocytes showed reduced expression of CX3CR1, CCR2, TLR4, and TLR5 with impaired migration towards MCP-1, reduced intracellular signaling (IFN-α induced pSTAT1, LPS induced IL-6) and increased expression of LPS induced IL-10. |
| Morton et al., 2019 (63) | HC (n = 10)  MAP (n = 12) MSAP (n = 1)  SAP (n = 2) | RAC | < 72 h | Blood | At < 24 h | (1) There were no differences in basal respiration, spare respiratory capacity, ATP turnover capacity, proton leak or non-mitochondrial respiration of monocytes between AP and HC.  (2) Monocyte basal ECARs were decreased in AP compared with HC but there were no differences in glycolytic reserve.  (3) Monocytes from AP exhibited higher PMA-induced OCRs and ECARs than HC. |
| Zhang et al., 2020 (16) | HC (n = 8)  AP (n = 21) | NA | NA | Blood | At < 24 h | (1) Circulating monocytes: AP > HC.  (2) PSGL-1 on monocytes: AP > HC. |
| Turunen et al., 2020 (64) | HC (n = 28)  MSAP (n = 6)  SAP (n = 3)  Sepsis (n = 14) | RAC | < 72 h | Blood | At < 48 h after admission and 2-4 and 5-8 d after the first sample | (1) Monocyte pNF-κB levels in response to TNF, LPS, and E. coli in patients with OD (SAP and sepsis complicated with OF patients) and to E. coli in patients without OD (MSAP patients) increased during follow-up but remained lower than HC.  (2) Constitutive pSTAT3 declined in monocytes of all patients during follow-up and in patients with OD reached the level of HC at the end of follow-up but still remained elevated in patients without OD than HC.  (3) Monocytes of all patients remained lower pSTAT3 and pSTAT1 levels in response to IL-6 throughout follow-up.  (4) Monocyte HLA-DR expression remained lower in AP than HC during follow-up though an increase trend in patients with OD. |
| Mubder et al., 2020 (65) | MAP (n = 198)  MSAP & SAP (n = 41) | RAC | NA | Blood | On admission, 1, and 2 d | Predicting MSAP/SAP: the cut-off value of LMR on admission, 1, and 2 d was < 2 with the sensitivity of 22.6%, 22.3%, 37.3%, and the specificity of 88.2%, 92.1%, 91.7% respectively. |
| Manohar et al., 2021 (17) | AP (n = 12)  RAP (n = 11) | NA | NA | Blood | On 1-5 d after admission | (1) CD14^+^CD16^-^ monocytes were highly abundant in blood from AP and RAP compared with the other 2 circulating monocyte subsets (nonclassical CD14^-^CD16^+^ and intermediate CD14^+^CD16^+^). Deeper profiling of the predominant CD14^+^CD16^-^ inflammatory monocytes identified 6 novel subsets based on differential expression of IL1β, IL27, and CD11c.  (2) Patients with gallstone had increased frequency of CD14^+^CD16^-^ and CD14^+^CD16^+^ monocytes compared with the patients with non-gallstone AP. Among the 6 novel CD14^+^CD16^-^ monocyte subsets, 3 were significantly increased in the gallstone AP group. |
| Susak et al., 2021 (66) | HC (n = 10)  MAP (n = 10)  MSAP (n = 68)  SAP (n = 19) | RAC | < 24 h | Blood | On admission | (1) Phagocytosis index of monocytes on admission: MSAP/SAP with septic complications < MSAP/SAP without septic complications < MAP < HC  (2) Monocytic ROS on admission: MSAP/SAP with septic complications > MSAP/SAP without septic complications > MAP > HC.  (3) Predicting infectious complications in MSAP/SAP: the AUC of monocyte phagocytosis reactivity reserve expressed as modulation coefficient values measured on admission at the cut-off value ≤ -24.8 was 0.84 with sensitivity of 76.2% and specificity of 83.6%. |
| Pian et al., 2021 (67) | Non-SAP (n = 119)  SAP (n = 50) | RAC | < 48 h | Blood | On 1, 3, and 7 d | (1) LMR: SAP < Non-SAP; 1 d: 1.44 (1.61) vs. 2.31 (2.23); 3 d: 1.63 (1.17) vs. 2.16 (2.20); 7 d: 2.27 (1.87) vs. 2.86 (2.39).  (2) LMR in SAP and non-SAP increased after 3 d. |
| Turunen et al., 2021 (68) | HC (n = 31)  MAP (n = 119) MSAP (n = 42) SAP (n = 13) | RAC | < 72 h | Blood | At < 24 h | (1) Monocytes in AP showed higher constitutive Tyr705 pSTAT3 levels, lower IL-6 stimulated pSTAT1 levels, lower IL-4 stimulated pSTAT6 levels, lower *E. coli* stimulated pNF-κB levels and lower LPS stimulated pAkt levels compared with HC.  (2) Predicting SAP at study entry: the AUC of constitutive Tyr705 pSTAT3 levels in monocytes was 0.725 (0.558-0.893).  (3) Predicting development of secondary infection: the AUC of constitutive Tyr705 pSTAT3 levels in monocytes was 0.662 (0.547-0.776). |
| Junare et al., 2021 (69) | MAP (n = 76) MSAP (n = 58) SAP (n = 26) | RAC | NA | Blood | At < 24 h | (1) LMR: SAP (1.57 ± 0.36) < MSAP (1.83 ± 0.33) < MAP (4.03 ± 1.39)  (2) Predicting ICU admission, OF, intervention, and mortality the AUC of LMR at the cut-off value ≤ 1.66, 2.1, 2.5, and 1.5 respectively was 0.863, 0.829, 0.846, and 0.805 with the sensitivity of 71.43%, 100%, 100%, and 66.67% and specificity of 91.2%, 58.65%, 54.84%, and 87.16%. |
| Khan et al., 2021 (70) | Non-SAP (n = 87)  SAP (n = 67) | SAP (CT severity score ≥ 7) | NA | Blood | On 1 d | (1) Monocyte counts (cells/μL): SAP (0.70 ± 0.30) > Non-SAP (0.56 ± 0.29)  (2) LMR: SAP (2.58 ± 2.28) < Non-SAP (3.75 ± 2.38).  (3) Predicting SAP: the adjusted odds ratio (multiple logistic regression) of LMR at the cut-off value ≤ 2.44 was 5.478. |
| Minkov et al., 2021(71) | HC (n = 24)  MAP (n = 39) MSAP (n = 24) SAP (n = 19) | RAC | NA | Blood | On admission, 2, and 5 d | (1) Percentage of CD14^+^HLA-DR^+^ monocytes: on admission, 2, and 5 d, AP < HC, SAP < MAP, AP with OF < AP without OF, AP with local complications < AP without local complications, AP with IPN < AP without IPN.  (2) Predicting SAP: the AUC of CD14^+^HLA-DR^+^ monocytes on admission, 2 or 5 d at the cut-off value ≤ 50.8%, 43.35% or 60.8% respectively was 0.728, 0.800 or 0.877 with the sensitivity of 72%, 84% or 82% and the specificity of 72%, 80% or 78%.  (2) Predicting unfavourable outcomes: the AUC of CD14^+^HLA-DR^+^ monocytes on 5 d at the cut-off value ≤ 38.3% was 0.944 with the sensitivity of 95% and the specificity of 93%. |
| Nalisa et al., 2021 (72) | HC (n = 6)  MAP (n = 14) MSAP (n = 11)  SAP (n = 4) | RAC | < 72 h | Blood | On 1, 3, 5, and 7 d after pain onset | (1) Classical monocyte subpopulations (CD14^+^CD16^-^) in the MSAP were 71.6% on 3 d, dropping to undetectable levels on 5 d, whereas those in SAP were 14.4% on 3 d and increased to 21.2% on 5 d.  (2) HLA-DR^+^ monocytes in the MSAP increased from 4.4% on 3 d to 47.1% on 5 d whereas those in SAP increased from 4.2% on 3 d to 13.5% on 5 d. |

Sampling time refers to time since admission unless otherwise stated. ∗ refers to the blood collected after the determination that the proportion of HLA-DR^+^ monocytes in the circulation was less than 80%. Abbreviations: HC, healthy controls; MAP, mild acute pancreatitis; SAP, severe acute pancreatitis; OF, organ failure; APACHE II, Acute Physiology and Chronic Health Evaluation II; LPS, lipopolysaccharide; TNF, tumor necrosis factor; IL, interleukin; OAC, original Atlanta classification; WBC, white blood cells; HLA-DR, human leukocyte antigen-DR; ICAM-1, intercellular adhesion molecule-1; OD, organ dysfunction; RFU, relative fluorescence unit; AUC, area under the receiver-operating-characteristic curve; MODS, multiple organ dysfunction syndrome; CRP, C reactive protein; PBMCs, peripheral blood mononuclear cells; GM-CSF, granulocyte-macrophage colony-stimulating factor; IFN, interferon; ICU, intensive care unit; TLR, Toll-like receptor; BMI, body mass index; TREM-1, triggering receptor expressed on myeloid cells; NF-κB, nuclear factor κB; MDP, N-acetylmuramyl-alanyl-Disoglutamine; STAT, signal transducer and activator of transcription; PMA, phorbol 12-myristate 13-acetate; ERK, extracellular signal-regulated kinase; MCP-1, monocyte chemoattractant protein-1; HO-1, heme oxygenase-1; SphK1, sphingosine kinase 1; RAC, revised Atlanta classification; LMR, lymphocyte-to-monocyte ratio; PD-L1, programmed cell death ligand 1; IPN, infected pancreatic necrosis; ANP, acute necrotising pancreatitis; CP, chronic pancreatitis; MDR, multi-drug resistant; MPV/PC, mean platelet volume-to-platelet count; NLR, neutrophil-to-lymphocyte ratio; MLR, monocyte-to-lymphocyte ratio; PLR, platelet-to-lymphocyte ratio; BISAP, Bedside Index of Severity in Acute Pancreatitis; G-CSF, granulocyte colony-stimulating factor; HTG, hypertriglyceridemic; ABP, acute biliary pancreatitis; HTG-AP, hypertriglyceridemic acute pancreatitis; poly I:C, polyinosilic:polycytidylic acid; ADAM17, a disintegrin and metalloproteinase 17; ATP, adenosine 5'-triphosphate; ECAR, extracellular acidification; OCR, oxygen consumption; PSGL-1, P-selectin glycoprotein ligand 1; RAP, recurrent acute pancreatitis; Tyr, tyrosine.

# SUPPLEMENTARY TABLE 3 | Effects of distinct lipid species, lipid modifications and dyslipidaemias on monocytes.

| **Lipids/Lipoproteins/Dyslipidaemias** | **Effects on monocytes** | **References** |
| --- | --- | --- |
| Saturated fatty acid, palmitate (C16:0) | ↑M1 polarisation | (73) |
| Monounsaturated fatty acid, oleate (C18:1) | ↑M2 polarisation | (73) |
| HDL | ↓activation | (74) |
| Apolipoprotein A-I | ↓activation | (74) |
| LDL-C | ↑number; ↑M1 polarisation | (75) |
| Low-oxidised LDL | ↑M1 polarisation | (76) |
| High-oxidised LDL | ↑M2 polarisation | (76) |
| Hypercholesterolaemia | ↑generation of classical monocytes | (77) |
| Hypertriglyceridaemia | ↑accumulation and extravasation of non-classical monocytes | (77) |

Abbreviations: HDL, high-density lipoprotein; LDL-C, low-density lipoprotein cholesterol; LDL, low-density lipoprotein.

# References

1. Goto M, Matsuno K, Yamaguchi Y, Ezaki T, Ogawa M. Proliferation kinetics of macrophage subpopulations in a rat experimental pancreatitis model. Archives of histology and cytology. 1993;56(1):75-82.

2. Satoh A, Shimosegawa T, Kimura K, Moriizumi S, Masamune A, Koizumi M, et al. Nitric oxide is overproduced by peritoneal macrophages in rat taurocholate pancreatitis: the mechanism of inducible nitric oxide synthase expression. Pancreas. 1998;17(4):402-11.

3. de Dios I, Perez M, de La Mano A, Sevillano S, Orfao A, Ramudo L, et al. Contribution of circulating leukocytes to cytokine production in pancreatic duct obstruction-induced acute pancreatitis in rats. Cytokine. 2002;20(6):295-303.

4. Liu HS, Pan CE, Liu QG, Yang W, Liu XM. Effect of NF-kappaB and p38 MAPK in activated monocytes/macrophages on pro-inflammatory cytokines of rats with acute pancreatitis. World J Gastroenterol. 2003;9(11):2513-8.

5. Dib M, Zhao X, Wang X, Andersson E, Drewsen G, Andersson R. Acute phase response in acute pancreatitis: a comparison with abdominal sepsis. Scand J Gastroenterol. 2003;38(10):1072-7.

6. Zhao X, Dib M, Wang X, Widegren B, Andersson R. Influence of mast cells on the expression of adhesion molecules on circulating and migrating leukocytes in acute pancreatitis-associated lung injury. Lung. 2005;183(4):253-64.

7. Shi C, Zhao X, Lagergren A, Sigvardsson M, Wang X, Andersson R. Immune status and inflammatory response differ locally and systemically in severe acute pancreatitis. Scand J Gastroenterol. 2006;41(4):472-80.

8. Rakonczay Z, Jr., Hegyi P, Dósa S, Iványi B, Jármay K, Biczó G, et al. A new severe acute necrotizing pancreatitis model induced by L-ornithine in rats. Crit Care Med. 2008;36(7):2117-27.

9. Perides G, Weiss ER, Michael ES, Laukkarinen JM, Duffield JS, Steer ML. TNF-alpha-dependent regulation of acute pancreatitis severity by Ly-6C(hi) monocytes in mice. J Biol Chem. 2011;286(15):13327-35.

10. Frossard JL, Lenglet S, Montecucco F, Steffens S, Galan K, Pelli G, et al. Role of CCL-2, CCR-2 and CCR-4 in cerulein-induced acute pancreatitis and pancreatitis-associated lung injury. J Clin Pathol. 2011;64(5):387-93.

11. Saeki K, Kanai T, Nakano M, Nakamura Y, Miyata N, Sujino T, et al. CCL2-induced migration and SOCS3-mediated activation of macrophages are involved in cerulein-induced pancreatitis in mice. Gastroenterology. 2012;142(4):1010-20.e9.

12. Guo F, Zheng S, Gao X, Zhang Q, Liu J. A novel acute necrotizing pancreatitis model induced by L-arginine in rats. Pancreas. 2015;44(2):279-86.

13. Schmidt AI, Seifert GJ, Lauch R, Wolff-Vorbeck G, Chikhladze S, Hopt UT, et al. Organ-specific monocyte activation in necrotizing pancreatitis in mice. The Journal of surgical research. 2015;197(2):374-81.

14. Yu E, Goto M, Ueta H, Kitazawa Y, Sawanobori Y, Kariya T, et al. Expression of area-specific M2-macrophage phenotype by recruited rat monocytes in duct-ligation pancreatitis. Histochemistry and cell biology. 2016;145(6):659-73.

15. Wu J, Zhang L, Shi J, He R, Yang W, Habtezion A, et al. Macrophage phenotypic switch orchestrates the inflammation and repair/regeneration following acute pancreatitis injury. EBioMedicine. 2020;58:102920.

16. Zhang X, Zhu M, Jiang XL, Liu X, Liu X, Liu P, et al. P-selectin glycoprotein ligand 1 deficiency prevents development of acute pancreatitis by attenuating leukocyte infiltration. World J Gastroenterol. 2020;26(41):6361-77.

17. Manohar M, Jones EK, Rubin SJS, Subrahmanyam PB, Swaminathan G, Mikhail D, et al. Novel Circulating and Tissue Monocytes as Well as Macrophages in Pancreatitis and Recovery. Gastroenterology. 2021;161(6):2014-29.e14.

18. Larvin M, Alexander DJ, Switala SF, McMahon MJ. Impaired mononuclear phagocyte function in patients with severe acute pancreatitis: evidence from studies of plasma clearance of trypsin and monocyte phagocytosis. Dig Dis Sci. 1993;38(1):18-27.

19. Liras G, Carballo F. An impaired phagocytic function is associated with leucocyte activation in the early stages of severe acute pancreatitis. Gut. 1996;39(1):39-42.

20. McKay CJ, Gallagher G, Brooks B, Imrie CW, Baxter JN. Increased monocyte cytokine production in association with systemic complications in acute pancreatitis. Br J Surg. 1996;83(7):919-23.

21. Salomone T, Boni P, Serra C, Morselli-Labate AM, Di Gioia AL, Romboli M, et al. The soluble interleukin-2 receptor, peripheral blood, and reticulocyte fractions in acute pancreatitis. Int J Pancreatol. 1996;20(3):197-203.

22. Richter A, Nebe T, Wendl K, Schuster K, Klaebisch G, Quintel M, et al. HLA-DR expression in acute pancreatitis. Eur J Surg. 1999;165(10):947-51.

23. Gotzinger P, Sautner T, Spittler A, Barlan M, Wamser P, Roth E, et al. Severe acute pancreatitis causes alterations in HLA-DR and CD14 expression on peripheral blood monocytes independently of surgical treatment. Eur J Surg. 2000;166(8):628-32.

24. Bhatnagar A, Wig J, Vaiphei K, Majumdar S. Intracellular cytokines in cells of necrotic tissue from patients with acute pancreatitis. Eur J Surg. 2001;167(7):510-7.

25. Bhatnagar A, Wig JD, Majumdar S. Expression of activation, adhesion molecules and intracellular cytokines in acute pancreatitis. Immunology letters. 2001;77(3):133-41.

26. Kylanpaa-Back ML, Takala A, Kemppainen E, Puolakkainen P, Kautiainen H, Jansson SE, et al. Cellular markers of systemic inflammation and immune suppression in patients with organ failure due to severe acute pancreatitis. Scand J Gastroenterol. 2001;36(10):1100-7.

27. Satoh A, Miura T, Satoh K, Masamune A, Yamagiwa T, Sakai Y, et al. Human leukocyte antigen-DR expression on peripheral monocytes as a predictive marker of sepsis during acute pancreatitis. Pancreas. 2002;25(3):245-50.

28. Mentula P, Kylanpaa-Back ML, Kemppainen E, Takala A, Jansson SE, Kautiainen H, et al. Decreased HLA (human leucocyte antigen)-DR expression on peripheral blood monocytes predicts the development of organ failure in patients with acute pancreatitis. Clin Sci (Lond). 2003;105(4):409-17.

29. Mentula P, Kylanpaa ML, Kemppainen E, Jansson SE, Sarna S, Puolakkainen P, et al. Plasma anti-inflammatory cytokines and monocyte human leucocyte antigen-DR expression in patients with acute pancreatitis. Scand J Gastroenterol. 2004;39(2):178-87.

30. Yu WK, Li WQ, Li N, Li JS. Mononuclear histocompatibility leukocyte antigen-DR expression in the early phase of acute pancreatitis. Pancreatology. 2004;4(3-4):233-43.

31. Rahman SH, Salter G, Holmfield JH, Larvin M, McMahon MJ. Soluble CD14 receptor expression and monocyte heterogeneity but not the C-260T CD14 genotype are associated with severe acute pancreatitis. Crit Care Med. 2004;32(12):2457-63.

32. Kylanpaa ML, Mentula P, Kemppainen E, Puolakkainen P, Aittomaki S, Silvennoinen O, et al. Monocyte anergy is present in patients with severe acute pancreatitis and is significantly alleviated by granulocyte-macrophage colony-stimulating factor and interferon-gamma in vitro. Pancreas. 2005;31(1):23-7.

33. Lindstrom O, Kylanpaa L, Mentula P, Puolakkainen P, Kemppainen E, Haapiainen R, et al. Upregulated but insufficient generation of activated protein C is associated with development of multiorgan failure in severe acute pancreatitis. Crit Care. 2006;10(1):R16.

34. Ho YP, Sheen IS, Chiu CT, Wu CS, Lin CY. A strong association between down-regulation of HLA-DR expression and the late mortality in patients with severe acute pancreatitis. Am J Gastroenterol. 2006;101(5):1117-24.

35. Li HG, Zhou ZG, Li Y, Zheng XL, Lei S, Zhu L, et al. Alterations of Toll-like receptor 4 expression on peripheral blood monocytes during the early stage of human acute pancreatitis. Dig Dis Sci. 2007;52(8):1973-8.

36. Mentula P, Kylanpaa ML, Kemppainen E, Repo H, Puolakkainen P. Early inflammatory response in acute pancreatitis is little affected by body mass index. Scand J Gastroenterol. 2007;42(11):1362-8.

37. Dabrowski A, Osada J, Dabrowska MI, Wereszczynska-Siemiatkowska U. Monocyte subsets and natural killer cells in acute pancreatitis. Pancreatology. 2008;8(2):126-34.

38. Ferat-Osorio E, Wong-Baeza I, Esquivel-Callejas N, Figueroa-Figueroa S, Duarte-Rojo A, Guzman-Valdivia-Gomez G, et al. Triggering receptor expressed on myeloid cells-1 expression on monocytes is associated with inflammation but not with infection in acute pancreatitis. Crit Care. 2009;13(3):R69.

39. Oiva J, Mustonen H, Kylänpää ML, Kyhälä L, Alanärä T, Aittomäki S, et al. Patients with acute pancreatitis complicated by organ failure show highly aberrant monocyte signaling profiles assessed by phospho-specific flow cytometry. Crit Care Med. 2010;38(8):1702-8.

40. Ho YP, Chiu CT, Sheen IS, Tseng SC, Lai PC, Ho SY, et al. Tumor necrosis factor-alpha and interleukin-10 contribute to immunoparalysis in patients with acute pancreatitis. Hum Immunol. 2011;72(1):18-23.

41. Habtezion A, Kwan R, Yang AL, Morgan ME, Akhtar E, Wanaski SP, et al. Heme oxygenase-1 is induced in peripheral blood mononuclear cells of patients with acute pancreatitis: a potential therapeutic target. Am J Physiol Gastrointest Liver Physiol. 2011;300(1):G12-20.

42. Li Q, Wang C, Zhang Q, Tang C, Li N, Li J. The role of sphingosine kinase 1 in patients with severe acute pancreatitis. Annals of surgery. 2012;255(5):954-62.

43. Oiva J, Mustonen H, Kylänpää ML, Kuuliala K, Siitonen S, Kemppainen E, et al. Patients with acute pancreatitis complicated by organ dysfunction show abnormal peripheral blood polymorphonuclear leukocyte signaling. Pancreatology. 2013;13(2):118-24.

44. Li JP, Yang J, Huang JR, Jiang DL, Zhang F, Liu MF, et al. Immunosuppression and the infection caused by gut mucosal barrier dysfunction in patients with early severe acute pancreatitis. Front Biosci-Landmrk. 2013;18(3):892-900.

45. Qin Y, Pinhu L, You Y, Sooranna S, Huang Z, Zhou X, et al. The role of Fas expression on the occurrence of immunosuppression in severe acute pancreatitis. Dig Dis Sci. 2013;58(11):3300-7.

46. Lin ZQ, Guo J, Xia Q, Yang XN, Huang W, Huang ZW, et al. Human leukocyte antigen-DR expression on peripheral monocytes may be an early marker for secondary infection in severe acute pancreatitis. Hepatogastroenterology. 2013;60(128):1896-902.

47. Zhang ML, Jiang YF, Wang XR, Ding LL, Wang HJ, Meng QQ, et al. Different phenotypes of monocytes in patients with new-onset mild acute pancreatitis. World J Gastroenterol. 2017;23(8):1477-88.

48. Li Y, Zhao Y, Feng L, Guo R. Comparison of the prognostic values of inflammation markers in patients with acute pancreatitis: a retrospective cohort study. BMJ open. 2017;7(3):e013206.

49. Pan T, Zhou T, Li L, Liu Z, Chen Y, Mao E, et al. Monocyte programmed death ligand-1 expression is an early marker for predicting infectious complications in acute pancreatitis. Crit Care. 2017;21(1):186.

50. Chen Y, Li M, Liu J, Pan T, Zhou T, Liu Z, et al. sPD-L1 Expression is Associated with Immunosuppression and Infectious Complications in Patients with Acute Pancreatitis. Scand J Immunol. 2017;86(2):100-6.

51. Sharma D, Jakkampudi A, Reddy R, Reddy PB, Patil A, Murthy HVV, et al. Association of Systemic Inflammatory and Anti-inflammatory Responses with Adverse Outcomes in Acute Pancreatitis: Preliminary Results of an Ongoing Study. Dig Dis Sci. 2017;62(12):3468-78.

52. Sendler M, Weiss FU, Golchert J, Homuth G, van den Brandt C, Mahajan UM, et al. Cathepsin B-Mediated Activation of Trypsinogen in Endocytosing Macrophages Increases Severity of Pancreatitis in Mice. Gastroenterology. 2018;154(3):704-18.e10.

53. Kuuliala K, Penttilä AK, Kaukonen KM, Mustonen H, Kuuliala A, Oiva J, et al. Signalling Profiles of Blood Leucocytes in Sepsis and in Acute Pancreatitis in Relation to Disease Severity. Scand J Immunol. 2018;87(2):88-98.

54. Zhang M, Ding L, Wang X, Hou J, Li M, Jiang Y, et al. Circulating CD14(+)CD163(+)CD115(+) M2 monocytes are associated with the severity of new onset severe acute pancreatitis in Chinese patients. International immunopharmacology. 2018;57:181-9.

55. Yu ZX, Chen XC, Zhang BY, Liu N, Gu Q. Association between HLA-DR Expression and Multidrug-resistant Infection in Patients with Severe Acute Pancreatitis. Curr Med Sci. 2018;38(3):449-54.

56. Djordjevic D, Rondovic G, Surbatovic M, Stanojevic I, Udovicic I, Andjelic T, et al. Neutrophil-to-Lymphocyte Ratio, Monocyte-to-Lymphocyte Ratio, Platelet-to-Lymphocyte Ratio, and Mean Platelet Volume-to-Platelet Count Ratio as Biomarkers in Critically Ill and Injured Patients: Which Ratio to Choose to Predict Outcome and Nature of Bacteremia? Mediators of inflammation. 2018;2018:3758068.

57. Qiu L, Zhou Y, Yu Q, Yu J, Li Q, Sun R. Decreased levels of regulatory B cells in patients with acute pancreatitis: association with the severity of the disease. Oncotarget. 2018;9(90):36067-82.

58. Kolber W, Kuśnierz-Cabala B, Maraj M, Kielar M, Mazur P, Maziarz B, et al. Neutrophil to lymphocyte ratio at the early phase of acute pancreatitis correlates with serum urokinase-type plasminogen activator receptor and interleukin 6 and predicts organ failure. Folia medica Cracoviensia. 2018;58(4):57-74.

59. Liu G, Tao J, Zhu Z, Wang W. The early prognostic value of inflammatory markers in patients with acute pancreatitis. Clinics and research in hepatology and gastroenterology. 2019;43(3):330-7.

60. Zhang R, Shi J, Zhang R, Ni J, Habtezion A, Wang X, et al. Expanded CD14(hi)CD16(-) Immunosuppressive Monocytes Predict Disease Severity in Patients with Acute Pancreatitis. J Immunol. 2019;202(9):2578-84.

61. Zheng J, Fan J, Huang C, Lu Y, Huang Z, Wang X, et al. Dynamic Detection of Monocyte Subsets in Peripheral Blood of Patients with Acute Hypertriglyceridemic Pancreatitis. Gastroenterology research and practice. 2019;2019:5705782.

62. Waller K, James C, de Jong A, Blackmore L, Ma Y, Stagg A, et al. ADAM17-Mediated Reduction in CD14(++)CD16(+) Monocytes ex vivo and Reduction in Intermediate Monocytes With Immune Paresis in Acute Pancreatitis and Acute Alcoholic Hepatitis. Front Immunol. 2019;10:1902.

63. Morton JC, Armstrong JA, Sud A, Tepikin AV, Sutton R, Criddle DN. Altered Bioenergetics of Blood Cell Sub-Populations in Acute Pancreatitis Patients. Journal of clinical medicine. 2019;8(12).

64. Turunen A, Kuuliala A, Penttilä A, Kaukonen KM, Mustonen H, Pettilä V, et al. Time course of signaling profiles of blood leukocytes in acute pancreatitis and sepsis. Scandinavian journal of clinical and laboratory investigation. 2020;80(2):114-23.

65. Mubder M, Dhindsa B, Nguyen D, Saghir S, Cross C, Makar R, et al. Utility of inflammatory markers to predict adverse outcome in acute pancreatitis: A retrospective study in a single academic center. Saudi journal of gastroenterology : official journal of the Saudi Gastroenterology Association. 2020;26(4):216-21.

66. Susak YM, Dirda OO, Fedorchuk OG, Tkachenko OA, Skivka LM. Infectious Complications of Acute Pancreatitis Is Associated with Peripheral Blood Phagocyte Functional Exhaustion. Dig Dis Sci. 2021;66(1):121-30.

67. Pian G, Li H, Piao Y. Clinical Significance of Inflammation Markers in Predicting the Severity of Acute Pancreatitis. Pancreas. 2021;50(2):201-5.

68. Turunen A, Kuuliala A, Mustonen H, Puolakkainen P, Kylänpää L, Kuuliala K. Blood Leukocyte Signaling Pathways as Predictors of Severity of Acute Pancreatitis. Pancreas. 2021;50(5):710-8.

69. Junare PR, Debnath P, Nair S, Chandnani S, Udgirkar S, Thange R, et al. Complete hemogram: simple and cost-effective in staging and predicting outcome in acute pancreatitis. Wien Klin Wochenschr. 2021;133(13-14):661-8.

70. Khan NA, Haider Kazmi SJ, Asghar MS, Singh M, Iqbal S, Jawed R, et al. Hematological Indices Predicting the Severity of Acute Pancreatitis Presenting to the Emergency Department: A Retrospective Analysis. Cureus. 2021;13(7):e16752.

71. Minkov G, Dimitrov E, Yovtchev Y, Enchev E, Lokova R, Halacheva K. Prognostic value of peripheral blood CD14+HLA-DR+ monocytes in patients with acute pancreatitis. Journal of immunoassay & immunochemistry. 2021;42(5):478-92.

72. Nalisa M, Nweke EE, Smith MD, Omoshoro-Jones J, Devar JW, Metzger R, et al. Chemokine receptor 8 expression may be linked to disease severity and elevated interleukin 6 secretion in acute pancreatitis. World journal of gastrointestinal pathophysiology. 2021;12(6):115-33.

73. Pararasa C, Ikwuobe J, Shigdar S, Boukouvalas A, Nabney IT, Brown JE, et al. Age-associated changes in long-chain fatty acid profile during healthy aging promote pro-inflammatory monocyte polarization via PPARγ. Aging Cell. 2016;15(1):128-39.

74. Murphy AJ, Woollard KJ, Hoang A, Mukhamedova N, Stirzaker RA, McCormick SP, et al. High-density lipoprotein reduces the human monocyte inflammatory response. Arteriosclerosis, thrombosis, and vascular biology. 2008;28(11):2071-7.

75. Stiekema LCA, Willemsen L, Kaiser Y, Prange KHM, Wareham NJ, Boekholdt SM, et al. Impact of cholesterol on proinflammatory monocyte production by the bone marrow. Eur Heart J. 2021;42(42):4309-20.

76. Seo JW, Yang EJ, Yoo KH, Choi IH. Macrophage Differentiation from Monocytes Is Influenced by the Lipid Oxidation Degree of Low Density Lipoprotein. Mediators of inflammation. 2015;2015:235797.

77. Rahman MS, Murphy AJ, Woollard KJ. Effects of dyslipidaemia on monocyte production and function in cardiovascular disease. Nature Reviews Cardiology. 2017;14(7):387-400.
